# Targeting an Initiator Allergen Provides Durable and Expansive Protection against House Dust Mite Allergy

**DOI:** 10.1021/acsptsci.2c00022

**Published:** 2022-08-12

**Authors:** Jihui Zhang, Jie Chen, Jonathan P. Richardson, Nicola-Jane Francis-Newton, Pei F. Lai, Kerry Jenkins, Meriel R. Major, Rebekah E. Key, Mark E. Stewart, Stuart Firth-Clark, Steven M. Lloyd, Gary K. Newton, Trevor R. Perrior, David R. Garrod, Clive Robinson

**Affiliations:** † Institute for Infection & Immunity, St. George’s, University of London, Cranmer Terrace, London SW17 0RE, United Kingdom; ‡ Domainex Ltd., Chesterford Research Park, Little Chesterford, Saffron Walden CB10 1XL, United Kingdom; § Faculty of Biology, Medicine and Health, University of Manchester, Manchester M13 9PL, United Kingdom

**Keywords:** house dust mite allergome, protease inhibitor, allergen, airway inflammation, eosinophil, Der p 1

## Abstract

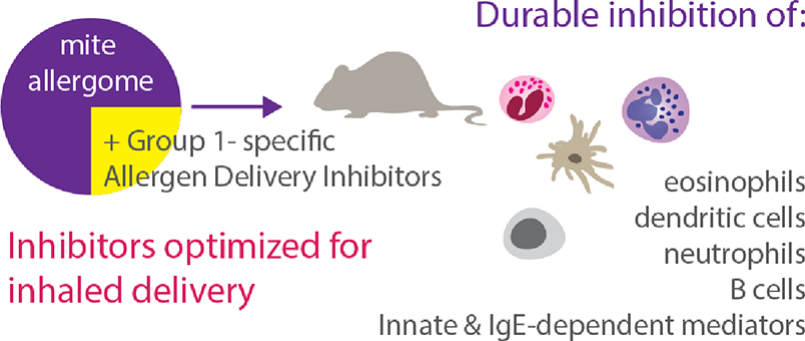

Whereas treatment of allergic diseases such as asthma relies largely on the targeting of dysregulated effector pathways, the conceptually attractive alternative of preventing them by a pharmaceutical, at-source intervention has been stymied until now by uncertainties about suitable targets and the challenges facing drug design. House dust mites (HDMs) are globally significant triggers of allergy. Group 1 HDM allergens, exemplified by Der p 1, are cysteine proteases. Their degradome has a strong disease linkage that underlies their status as risk and initiator allergens acting directly and through bystander effects on other allergens. Our objective was to test whether target-selective inhibitors of group 1 HDM allergens might provide a viable route to novel therapies. Using structure-directed design to optimize a series of pyruvamides, we undertook the first examination of whether pharmaceutically developable inhibitors of group 1 allergens might offer protection against HDM exposure. Developability criteria included durable inhibition of clinically relevant signals after a single aerosolized dose of the drug. The compounds suppressed acute airway responses of rats and mice when challenged with an HDM extract representing the HDM allergome. Inhibitory effects operated through a miscellany of downstream pathways involving, among others, IL-33, thymic stromal lymphopoietin, chemokines, and dendritic cells. IL-13 and eosinophil recruitment, indices of Th2 pathway activation, were strongly attenuated. The surprisingly expansive benefits arising from a unique at-source intervention suggest a novel approach to multiple allergic diseases in which HDMs play prominent roles and encourage exploration of these pharmaceutically developable molecules in a clinical setting.

Historically, there has been conjecture that improved management of allergic diseases could come from apex interventions acting at-source on root cause disease triggers, but the realization of this hypothesis has been stymied by uncertainty surrounding whether effective targets exist and their chemical tractability.^1^ Allergy to house dust mites (HDMs) offers an opportunity to examine this concept because HDMs are themselves not only important triggers of disease, but they also promote polysensitization to unrelated allergens.^1,2^ To assess the feasibility of an at-source intervention, it is necessary to understand which allergens constitute a biologically significant target with drug development opportunities. Using pharmaceutically developable inhibitors in tandem with effector cell and mediator biosignatures that are clinically validated by mechanism-based therapies, we examined whether the inhibition of the intrinsic bioactivity of disease initiator/risk allergens could be a potentially exploitable approach.^1,3−6^

The allergome of HDMs comprises >30 diverse allergen groups.^1,2^ Group 1 HDM allergens are homologous C1 cysteine proteases^7^ whose bioactivity and immunogenicity grant them status as autonomous initiator allergens with high disease linkage.^2,8,9^ This initiator function raises the possibility that their inhibition might yield expansive benefits by blocking the effects of unrelated allergens from HDMs or other sources. Human exposure to HDM allergens occurs through their presence within excreted fecal pellets that are of a respirable diameter or can contact the skin. Upon impaction of the airway lining, their contents are released into the airway surface liquid (ASL) that, through its reducing agent content, favors the catalytic competence of cysteine proteases.^10−13^ This proteolytic activity of group 1 HDM allergens is salient to an allergic diathesis.^1,8,14−18^ An unexpected facet of their degradome is prothrombinase activity.^1,5,19^ Thrombin generated in airway epithelial cells by this activity stimulates protease activated receptors (PARs)-1 and -4 and epidermal growth factor receptor (EGFR)-dependent signaling to generate reactive oxidant species (ROS) that entrain the expression of cytokines through effects on histones, redox-dependent transcription factors, and signaling proteins.^20,21^ Essential to this sequence is the recruitment of a cellular prothrombinase via the activity of a disintegrin and metalloprotease 10 (ADAM 10)^5,22^ that leads to the generation of endogenous ligands of Toll-like receptor (TLR) 4.^5^ TLR4 signaling is central to allergic sensitization; altered expression and polymorphisms in both the receptor and its ligand bioprocessing pathways are also disease risks.^16,23−25^ In mice, epithelial TLR4 signaling presages the activation of dendritic antigen presenting cells (DCs) and allergic sensitization of the lungs to HDMs^26^ that is further modulated by TLR4 on repeated allergen exposure.^27^ The protease-dependent ability of group 1 HDM allergens to generate endogenous TLR4 ligands thus provides a fundamental linkage between TLR4 and the prominent role of group 1 HDM allergens in allergic sensitization of the lungs.^1,5,8^

Alongside these events, group 1 HDM allergens directly and indirectly increase the permeability of epithelial barriers.^1,9,28,29^ These mechanisms facilitate transepithelial allergen delivery of *any* allergen, thus increasing the probability of contact with dendritic antigen presenting cells (DCs) and the reinforcement of allergy. This process may be augmented by ADAM 10 that, beyond the actions outlined above and its regulation of IgE production, is a sheddase for E-cadherin.^30^ E-cadherin is a component of epithelial adherens junctions, but separately from this role, it prevents interleukin (IL)-5 and IL-13 release from type 2 innate lymphoid cells (ILC2) by ligation of killer cell lectin-like receptor sub-family G, member 1.^31^ Thus, untethering of ILC2 cells by group 1 HDM allergens and the E-cadherin shedding action of ADAM 10 is a likely innate checkpoint for IL-5 and IL-13 production in allergy progression.

Der p 1 is the group 1 allergen from *Dermatophagoides pteronyssinus* and is commonly studied as a representative of other group 1 HDM allergens with which it is homologous.^1,2^ Generic inhibitors of cysteine proteases prevent Der p 1 from triggering the development of Der p 1-specific IgE in experimental models, but because this finding is based on tools with poor pharmacological credentials, weak potency, and low selectivity, it is not known whether the suppression is due to the direct inhibition of the Der p 1 or through off-target effects in the host.^1^ Moreover, while the risk/initiator allergen status of group 1 suggests that its inhibition might confer protection against other HDM allergen groups, demonstration that the development of Der p 1-specific IgE is inhibited following exposure to purified Der p 1 and a generic inhibitor does not address this concept of an expansive, broad-spectrum protection against unrelated allergens. To resolve these matters, novel pyruvamide inhibitors of group 1 HDM allergens have been created by structure-based drug design.^1,3,4^ Compounds designed against this target have been designated ″allergen delivery inhibitors″ (ADIs) because of their protective effects on airway epithelium. Using model cell systems, we have found that ADIs prevent TLR4-dependent ROS generation by HDM extracts, suggesting that inhibition of multiple pathways that are reliant on TLR4 ligation and redox-dependent gene expression should result.^5^ Now, using clinically developable representatives from this series of new molecular entities (NMEs), our aim was to test whether an at-source intervention directed against an initiator/risk allergen could modify both acute innate and allergic responses to the wider HDM allergen repertoire.

## Results and Discussion

The rationale behind this work is a desire to improve the treatment of allergy.^1^ While the quest to attack root causes of allergy is long-standing, the pursuit of a developable pharmacological solution must confront significant obstacles.^1,6,8^ Group 1 HDM allergens are exploitable candidates in this enterprise because they are autonomous initiator allergens that facilitate polysensitization to other mite and non-mite allergens of diverse structures and functions.^1,2^ Conveniently for NME design, group 1 HDM allergens couple strong disease linkage with the potentially advantageous safety profile afforded by a non-human target.^1,3,6^ Moreover, because Der p 1 inactivates serpins, an incidental benefit is the inhibition of HDM serine peptidase allergens through the protection of airway antiprotease defenses by ADI compounds.^1,6,8^

At the inception of this program, the ability to obtain durable protection against the HDM allergome from a single dose of drug was unanticipated, and our speculation was that ADIs would probably exert efficacy only upon chronic treatment. However, reappraisal of this opinion was necessary because a forerunner chemical series of the pyruvamides described herein boosted the possibility that ADIs could have unanticipated benefits in acute allergy.^6^ Although these forerunner aminoketones lacked the credentials to prove this concept, they were significant in providing the encouragement to design an entirely new series with the properties that could. The pyruvamides that are the focus of this paper resulted from this design and optimization campaign and now instantiate the acute benefits of ADIs in preclinical models. Focused analogue libraries were used to optimize properties using Der p 1 as the target archetype, the library described herein being used to identify attributes that combine target potency and durability of action. For a detailed study, we selected a pair of pyruvamides that are differentiated by the pharmacokinetic behavior while exhibiting a similar target potency (Table 1).

**Table 1:**
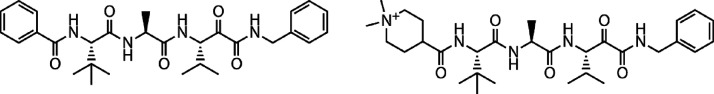
Property Profiles of Pyruvamides **1** and **2** as Determined by Techniques Described in Materials and Methods^a^

	compound 1	compound 2
target potency (*K*_i_)	4.5 nM	1 nM
log D	3.2	–0.9
thermodynamic solubility	84 μM	>1.6 mM
permeability (*P*_app_)	6.2 × 10^–6^ cm s^–1^	ND
cell stability (L2 cells; macrophages)	100; 100	100;100
plasma stability (% remaining in 2 h)	100 (rat); 100 (human)	100 (rat); 100 (human)
plasma protein binding (rat; human, %)	96; 99.6	61;63
oral bioavailability (rat, %)	39 (fasted); 33.5 (fed)	0.6 (fed)
*C*_max_ (5 mg/kg p.o. dose, rat)	41 nM (free fraction; fasted)	5 nM (free fraction; fasted)
volume of distribution (L/kg, rat p.o.)	0.9	0.3
clearance (mL/min/kg, rat p.o.)	27 (fasted)	19 (fasted)
half-life (h, rat p.o.)	2.1 (fasted), 2.9 (fed)	0.3 (fed)
hepatocyte half-life (min)	118 (rat); 181 (human)	∞ (rat); ∞ (human)
hepatocyte intrinsic clearance (mL/min/kg)	28.2 (rat); 11.7 (human)	0 (rat); 0 (human)

a Details of selectivity profiles are presented elsewhere.^3^

Compound **1** is a neutral molecule, whereas compound **2** is a quaternary amine. Each is a potent, reversible, and selective inhibitor of group 1 HDM allergens but differs in their approach to optimizing lung retention while mitigating adverse events from either local or systemic effects. The target allergen group triggers responses by extracellular molecular recognition, notably via PARs and tight junction adhesion proteins,^1,2,8,28^ so no requirement exists for inhibitors to be cell-permeant, enabling an option for enhancing lung retention and extracellular effects by forming quaternary amines, such as compound **2**. In contrast, the neutral and absorbable compound **1** sought lung retention through moderate lipophilicity.

### Studies in Rats Sensitized to the HDM Allergome

Sensitization was associated with elevated serum IgE that comprised IgE reactive with the HDM extract generally and with Der p 1 specifically. HDM-directed IgG_2a_ was generated, although total IgG_2a_ was unaffected (Supporting Information Figure S1A–E). Sensitized animals developed a clear eosinophil response to the aerosol challenge (Supporting Information Figure S1F).

Aerosol challenge with the HDM extract increased inflammatory cells in the bronchoalveolar lavage (BAL) fluid. This was characterized by an influx of neutrophils that was resolved by 72 h, whereas the appearance of eosinophils was gradual and sustained (Supporting Information Figure S1G–I). The rising trend of BAL monocytes/macrophages was generally not significant (Supporting Information Figure S1J).

### Single Doses of ADI Compounds Suppress Responses to the HDM Extract

Our initial focus was to investigate the efficacy of selected ADIs in influencing the recruitment of eosinophils following HDM challenge because these cells demarcate an important clinical phenotype in allergic asthma. Furthermore, they drive key stages of type 2 inflammation and the development of persistent airflow obstruction in people with asthma. Accordingly, most experiments used a BAL sampling time optimized for this readout. We electively chose an acute allergen provocation model with an extract of mixed allergens rather than purified Der p 1 because this provides a rigorous test of whether broad protection could be achieved against a representation of the HDM allergome.

**Figure 1:**
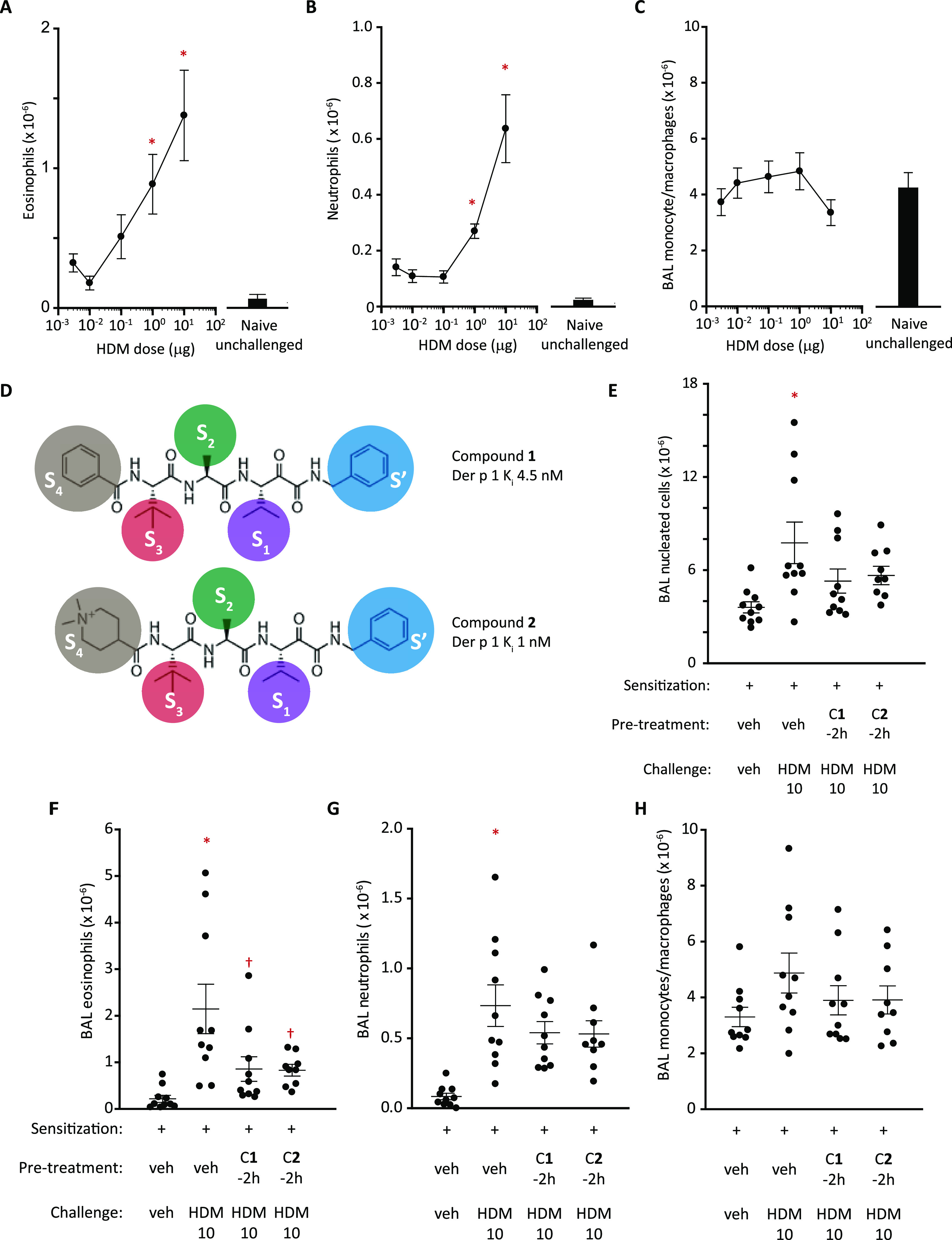
HDM challenge in rats sensitized to the HDM allergen extract and its modification by ADI compounds. (A–C) Changes in BAL eosinophil, neutrophil, and monocyte/macrophage composition assessed by light microscopy 48 h following i.t. aerosol challenge with the HDM extract at a range of doses. Data are shown as mean ± S.E. from 10 animals per dose level. *P < 0.001–0.05 *vs* unchallenged animals. Black columns depict control responses in unsensitized, unchallenged animals. (D) Compounds **1** and **2** showing functional groups and their predicted protease subsite interactions (S′–S4) with Der p 1 used as the archetype for other group 1 HDM allergens. (E–H) Effects of ADI compounds **1** or **2** on BAL cell composition following i.t. aerosol challenge with the HDM extract (HDM 10 with 10 μg Der p 1 content). Animals were pretreated with ADIs 2 h prior to the allergen challenge (dose by i.t. aerosol 15 μg/kg for compound **1** and 46 μg/kg for compound **2**). Data are individual responses with mean ± S.E. depicted by whiskers with *n* = 10 per treatment group. Note that in the compound **2** group, one animal was euthanized for welfare reasons following the challenge. *P < 0.001 *vs* vehicle (veh) challenge. ^†^*P* < 0.01–0.05 *vs* HDM 10 challenge.

A dose–response relationship existed for the numbers of eosinophils and neutrophils recovered by BAL following challenge (Figure 1A,B), but monocytes/macrophages were unaffected (Figure 1C), generally consistent with other data (Supporting Information Figure S1J). Remarkably, a single dose of compound **1** or **2** (Figure 1D) administered 2 h before HDM challenge attenuated the changes in BAL cells (Figure 1E), primarily due to eosinophil suppression (Figure 1F). The trend toward blunting of neutrophil responses was not significant, possibly due to the suboptimal sampling time for these cells (Figure 1G). No effects on monocytes/macrophages were seen (Figure 1H). Thus, acute effects of HDM allergen extracts that contain clinically important allergens unrelated to the group 1 target are suppressed by group 1-specific inhibition. In this regard, compounds **1** and **2** had similar pharmacodynamics despite their markedly different pharmacokinetics and physicochemistry. Unlike compound **2**, where quaternization restricts the molecule to the airways, **1** might be expected to show a less persistent action due to transepithelial absorption, but this was not evident within 2 h. Next, using compound **2** for exemplification, we verified that Der p 1 was a major activator of these responses in sensitized animals, and as expected, the changes in eosinophils were inhibited (Supporting Information Figure S1K,L). Particularly in this study, compound **2** also inhibited a neutrophil response. The interstudy variability in neutrophil data was a recurring feature, likely resulting from the sampling time used in most of the studies described herein being suboptimal for neutrophils.

### The Durable Action of ADIs Accompanies the Inhibition of Sentinel Biosignatures

**Figure 2:**
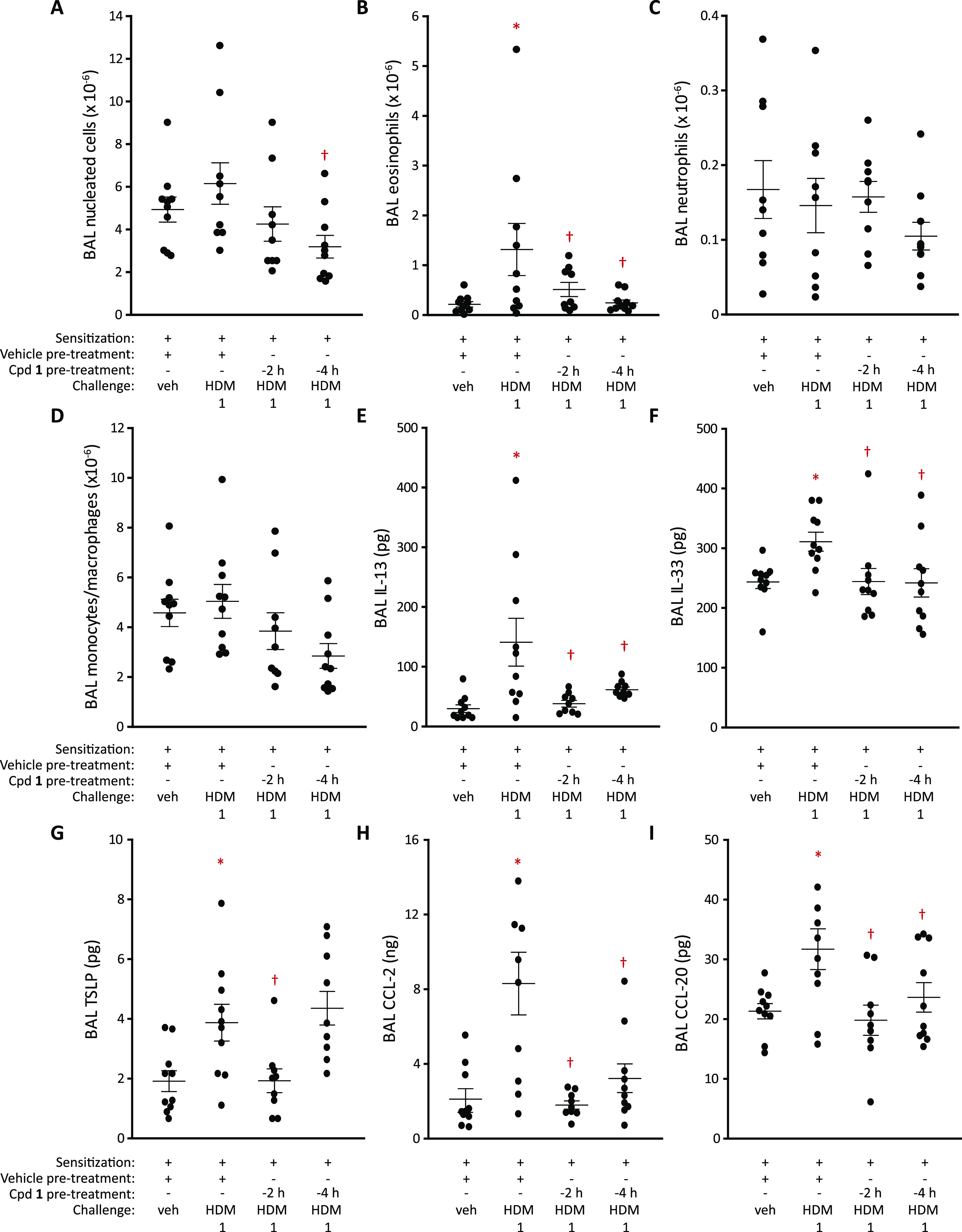
Modification of cell and mediator responses to the HDM challenge in sensitized rats by compound **1**. (A–D) Effect of dosing compound **1** by i.t. aerosol (doses as in Figure 1) 2 or 4 h prior to challenge with the HDM extract (equivalent to 1 μg Der p 1; i.t. aerosol) on the cellular composition of BAL fluid at 48 h as assessed by light microscopy. (E–I) BAL mediator responses 48 h after the HDM extract challenge. Data are individual responses with mean ± S.E. depicted by whiskers. *P < 0.05 *vs* vehicle challenge. ^†^*P* < 0.05 *vs* HDM 1 challenge.

Encouraging data prompted a deeper exploration of the durability of compound **1**. Figure 2A–D depicts its effects when administered at different times before the HDM challenge. In agreement with initial findings, it suppressed BAL eosinophil responses similarly when administered 2 or 4 h before challenge (Figure 2B). No effect was apparent on neutrophil recruitment due to the resolution of the positive control response (Figure 2C). The HDM challenge was associated with elevated levels of IL-13, IL-33, thymic stromal lymphopoietin (TSLP), C-C chemokine ligand-2 (CCL-2), and CCL-20 in BAL fluid, and all were suppressed by compound **1**, except for TSLP, when dosed 4 h before challenge (Figure 2E–I). Collectively, these data suggest that pyruvamide **1** has an encouraging persistence of action on clinically relevant cellular and molecular readouts that belies the absence of the quaternary amine moiety.

**Figure 3:**
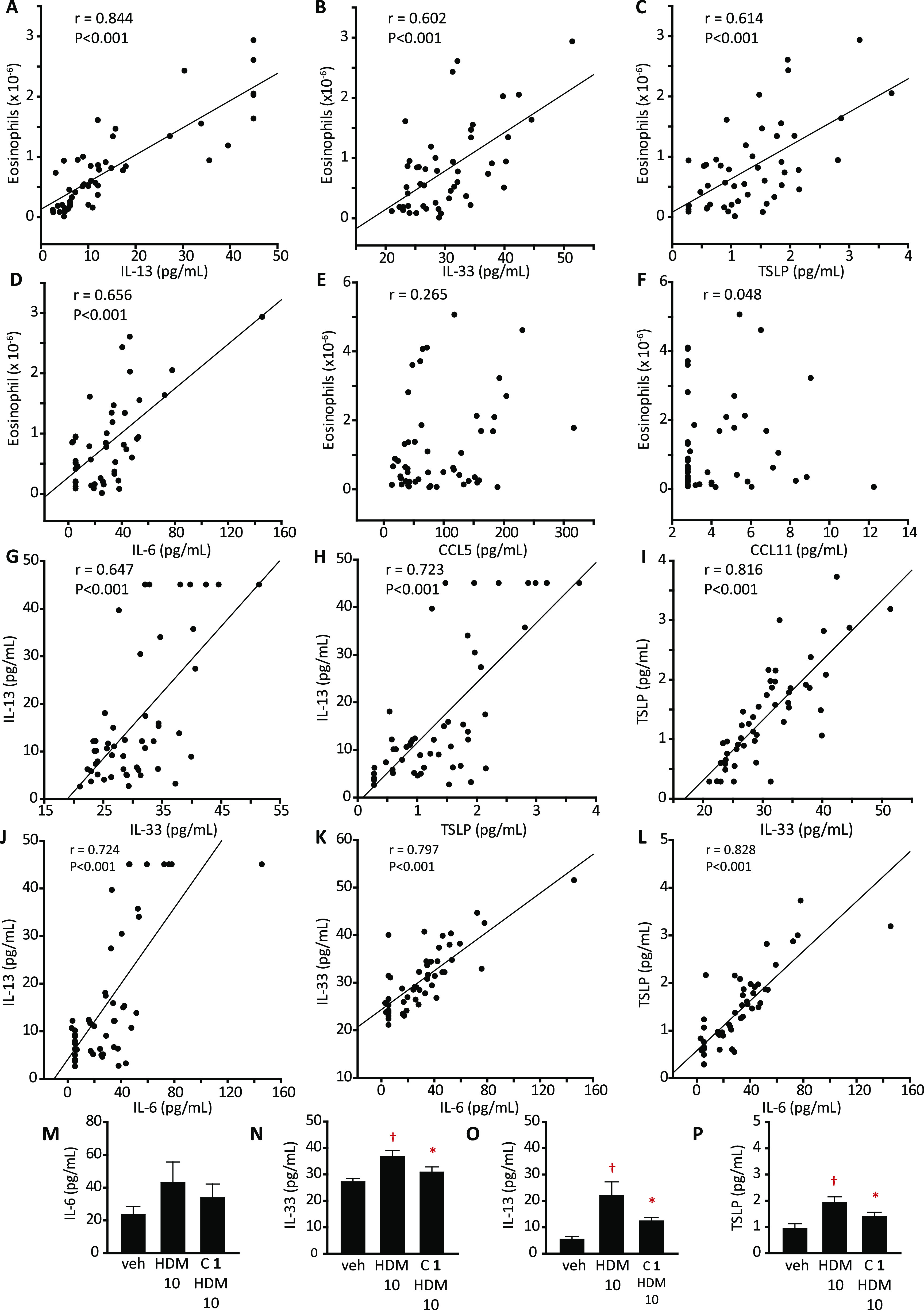
Eosinophil counts determined by light microscopy and cytokine/chemokine in BAL fluid from BN rats and inhibition by compound **1**. (A–F) Relationships between eosinophil numbers and cytokines/chemokines. (G–L) Comparison of relationships between individual cytokines and chemokines. (M–P) Modulation of BAL cytokine levels by compound **1** (15 μg/kg, i.t. aerosol) administered 2 h prior to the aerosol challenge with the HDM allergen extract (HDM 10, equivalent to 10 μg Der p 1). Data are mean ± S.E. in 10 animals per group. ^†^*P* < 0.01 *vs* veh, *P < 0.05 *vs* HDM 10.

Examination of relationships between mediators and cell numbers revealed correlations between BAL eosinophils and IL-13, IL-33, TSLP, and IL-6 but not CCL5 and CCL11 (Figure 3A–F). BAL concentrations of IL-13, IL-33, TSLP, and IL-6 were correlated (Figure 3G–L). Compound **1** was further evaluated against a stronger challenge, confirming the inhibitory effects on IL-33, IL-13, and TSLP, whereas IL-6 responses were unaffected (Figure 3M–P). Concentrations of CCL2 and CCL20 in BAL were correlated but not to the cell populations studied (Supporting Information Figure S2A–G). Weak correlations existed with IL-6 (Supporting Information Figure S2 H–J).

### Innate Responses in Rats

**Figure 4:**
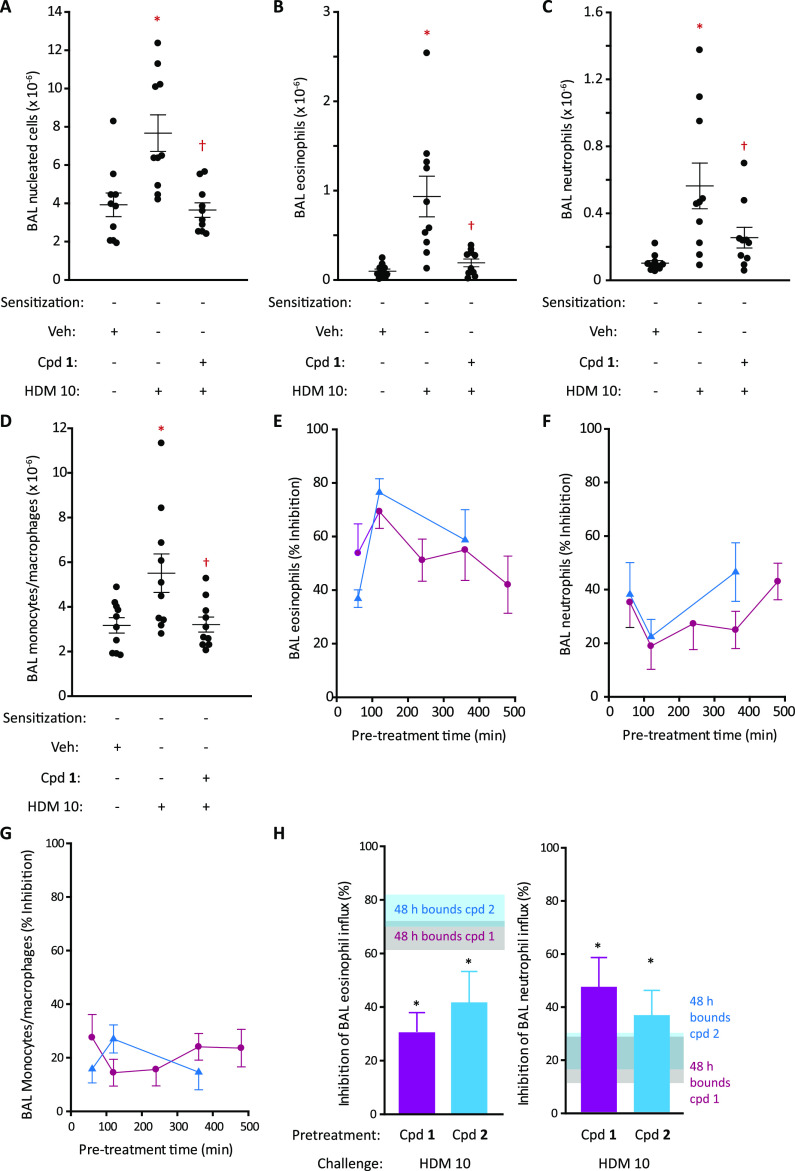
ADI compounds **1** and **2** attenuate innate responses to the HDM challenge in unsensitized BN rats. (A–D) Effect of dosing compound **1** (15 μg/kg; i.t. aerosol) 2 h prior to the HDM extract challenge (HDM 10, equivalent to 10 μg Der p 1; i.t. aerosol) on the cellular composition of BAL fluid at 48 h. Data are individual responses with mean ± S.E. depicted by whiskers. Cell numbers were counted by light microscopy. *P < 0.001–0.05 *vs* vehicle challenge. ^†^*P* < 0.001–0.05 *vs* HDM 10 challenge. (E–G) Relationships between pretreatment interval for a single i.t. dose of either ADI compound **1** (magenta circles, 15 μg/kg) or **2** (blue triangles, 46 μg/kg) and changes in BAL cell composition 48 h after allergen challenge. Inhibition of eosinophil responses was *P* < 0.001 for all pretreatment intervals for both compounds. For neutrophils, with compound **2**, the effects were significant at 60 min (*P* < 0.01) and 360 min (*P* < 0.01). For monocytes/macrophages, inhibition by compound **1** was significant at 60 min (*P* < 0.05), whereas with compound **2**, the dosing 120 min prior to challenge was significant (*P* < 0.01). (H) Inhibition of eosinophil and neutrophil responses 24 h after the HDM extract challenge. Compounds **1** (15 μg/kg, magenta bars) and **2** (46 μg/kg, blue bars) were dosed 2 h prior to the HDM extract. Horizontal gray (compound **1**) or blue (compound **2**) shaded boxes show the S.E. mean bounds of inhibition for each compound at 48 h, with the gray–blue merged zone indicating where these bounds overlap. *P < 0.05 *vs* control challenge. In E–H, data are shown as mean ± S.E. from 10 animals.

Inhibition of sentinel innate response signals by compound **1** (Figure 2E–I) led us to investigate events in HDM-naive rats. Figure 4A–D shows that the HDM challenge increased the cellularity of BAL fluid, including significant elevations in monocytes/macrophages. Prior exposure to compound **1** suppressed these, including changes in neutrophil and monocyte/macrophage numbers (Figure 4A–D).

### Multiple Approaches to Lung Retention Achieve Protection against HDM

To understand the factors governing the duration of action of pyruvamides, we compared compounds **1** and **2** with a focused analogue library (Figure 4E–G and Supporting Information Figure S3). For **1** and **2**, while peak effects on eosinophils occurred when compounds were administered 2 h before the challenge, there was substantial inhibition with even 6–8 h separation (Figure 4E). What dictates the onset of protection is unknown, but distribution and partitioning within ASL and the apical airway epithelium are plausible leading factors. Similarly, multiple influences likely determine why inhibition (>60% at peak), while impressive in an acute challenge with the HDM allergome surrogate, was incomplete for both compounds. The dynamics of interaction between the drug dispersed in the airway and inhaled allergen might simply allow a fraction of the target to evade immediate inhibition. Alternatively, the innate cellular response that remains in the presence of **1** or **2** may be due to other allergens from the HDM repertoire whose roles are independent from, but evidently subsidiary to, those of group 1. Regardless, the inhibition by the ADI compounds was both striking and enduring.

Compounds **1** and **2** had complex effects on BAL neutrophils in that short or long pretreatment intervals were inhibitory, but intervening changes were insignificant (Figure 4F). A modest inhibition of monocytes/macrophages occurred at shorter pretreatment times for both compounds (Figure 4G). To better characterize the effects on neutrophils, BAL was performed 24 h after the HDM extract challenge, whereupon a clear inhibition of the response was revealed (Figure 4H).

**Figure 5:**
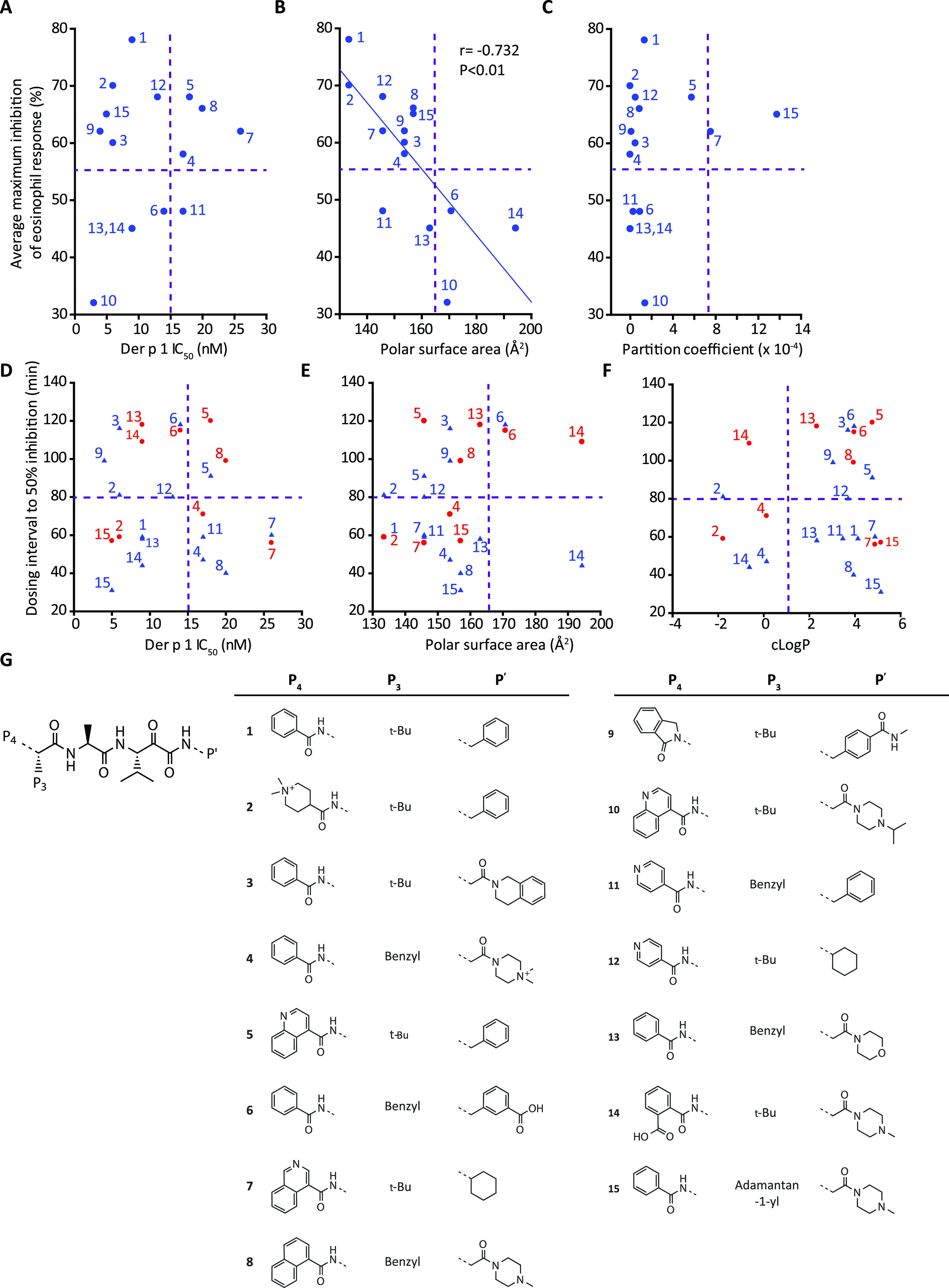
Characteristics of pyruvamide ADIs and duration of protection against HDM challenge. (A–C) Quadrant plots showing relationships between the maximum inhibition of the eosinophil response and inhibitory potency, polar surface area, and partition coefficient. (D–F) Plots of potency, polar surface area, and cLogP as functions of the time taken to achieve 50% inhibition of eosinophil (blue triangles) or neutrophil (red circles). In A–F, each symbol represents the average biological response from groups of 10–12 animals. (G) Structures of compounds **1**–**15** used in these studies.

Durability of inhibition studies using the focused library (compounds **1**–**15**) (Figure 4 and Supporting Information Figure S3) had enabled us to explore the properties that blended desirable attributes required of clinical development candidates (Figure 5). These data support the selection of compounds **1** and **2** for developability assessment (Table 1) because they performed well *in vivo.* Others (e.g., compound **10**), while potent against the target molecule *per se*, performed less satisfactorily *in vivo* (Figure 5A). Conversely, compounds **4**, **5**, **7**, **8**, and **11** (in the right-hand quadrants of Figure 5A), while less potent than others *in vitro*, were effective *in vivo.* An inverse linear relationship exists between the topological polar surface area (PSA) of inhibitors and the maximum effect on eosinophils (Figure 5B) showing that compounds of greater PSA were less satisfactory choices *in vivo* than implied simply by their *in vitro* potency. In contrast, computed partition coefficient (cLog P) was a less discerning indicator, with some compounds (notably **7** and **15**) separated from others while having useful *in vivo* activity (Figure 5C). To aid understanding, we estimated the temporal separation between drug dosing and HDM challenge required to achieve 50% inhibition of the eosinophil response. Compounds **1** and **2** could be differentiated by the former being faster in onset when ranked by IC_50_, PSA, and cLog P (Figure 5D–F). Compound **5** represented a different option from **4**, **7**, **8**, and **11** in having a slower onset despite other similarities. Likewise, compound **4** was distinguishable when judged by cLog P (Figure 5F). Generally, while faster onset could be obtained from compounds with a low PSA or high cLog P, there is additional complexity as illustrated by compounds **5** and **2**. Therefore, compounds **1** and **2** embody desirable characteristics, endorsing their selection for detailed study and developability evaluation (Figure 5A,D–F, Table 1).

### Innate and Acquired Responses in Mice

**Figure 6:**
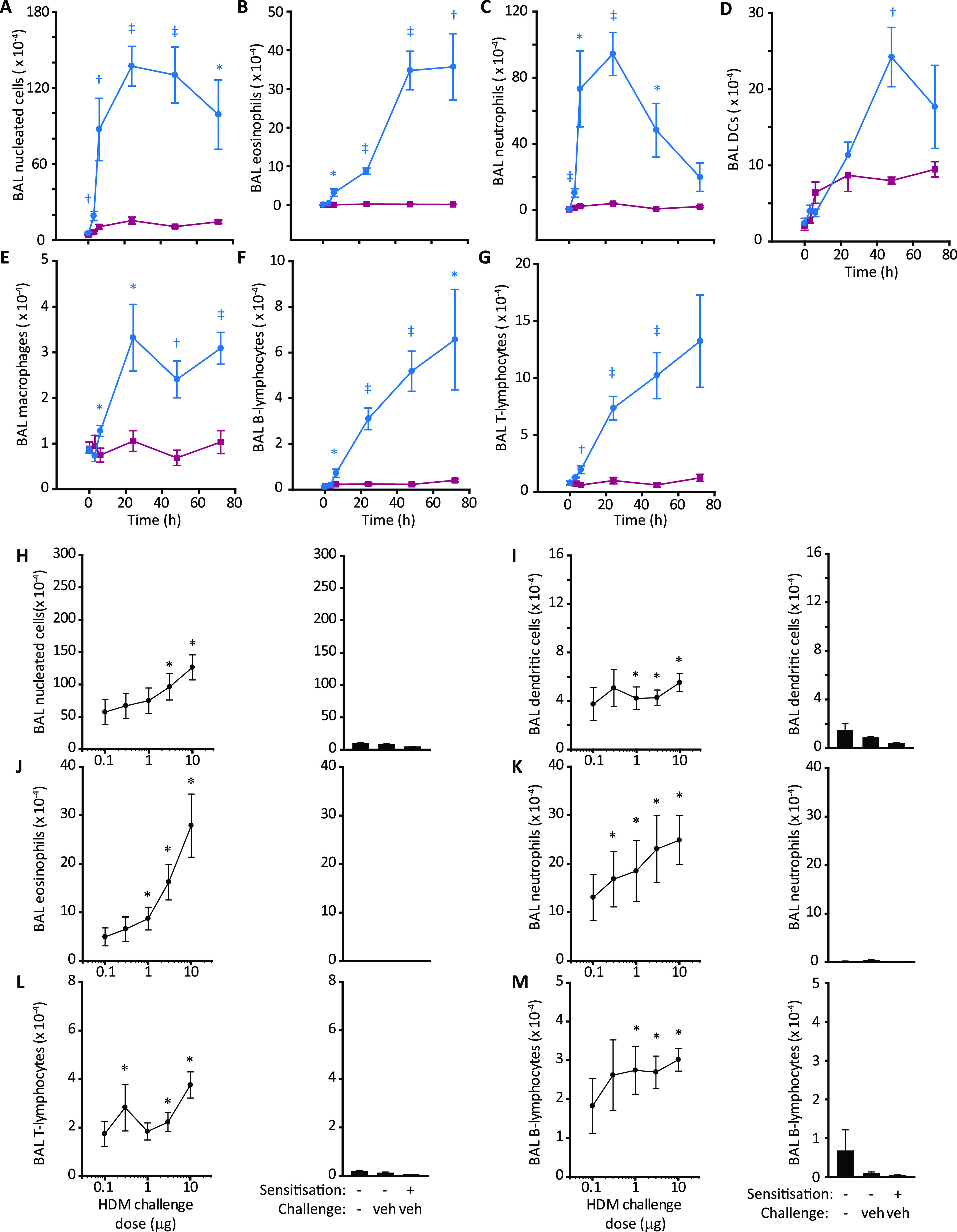
Time course and dose-dependency of cellular responses to the HDM extract in Balb/c mice. (A–G) BAL cell counts for individual cell types enumerated by flow cytometry. Blue lines and circles show animals immunized with the HDM allergen extract and subsequently challenged with HDM (i.t. aerosol, equivalent to 10 μg Der p 1). Magenta lines and squares depict data for HDM sensitized animals challenged with the vehicle. Data are shown as mean ± S.E. in five animals per group. *P < 0.05, ^†^*P* < 0.01, ^‡^*P* < 0.001 vs corresponding vehicle challenge time point. (H–M) Analysis of cell counts 48 h after the challenge. The left-hand side of each panel shows dose–response data as mean ± S.E. from groups of 10 animals. *P < 0.05 *vs* unchallenged, unsensitized mice. Doses are expressed as the quantity of Der p 1 delivered by aerosol to the airways. The right-hand side of each panel depicts control data.

**Figure 7:**
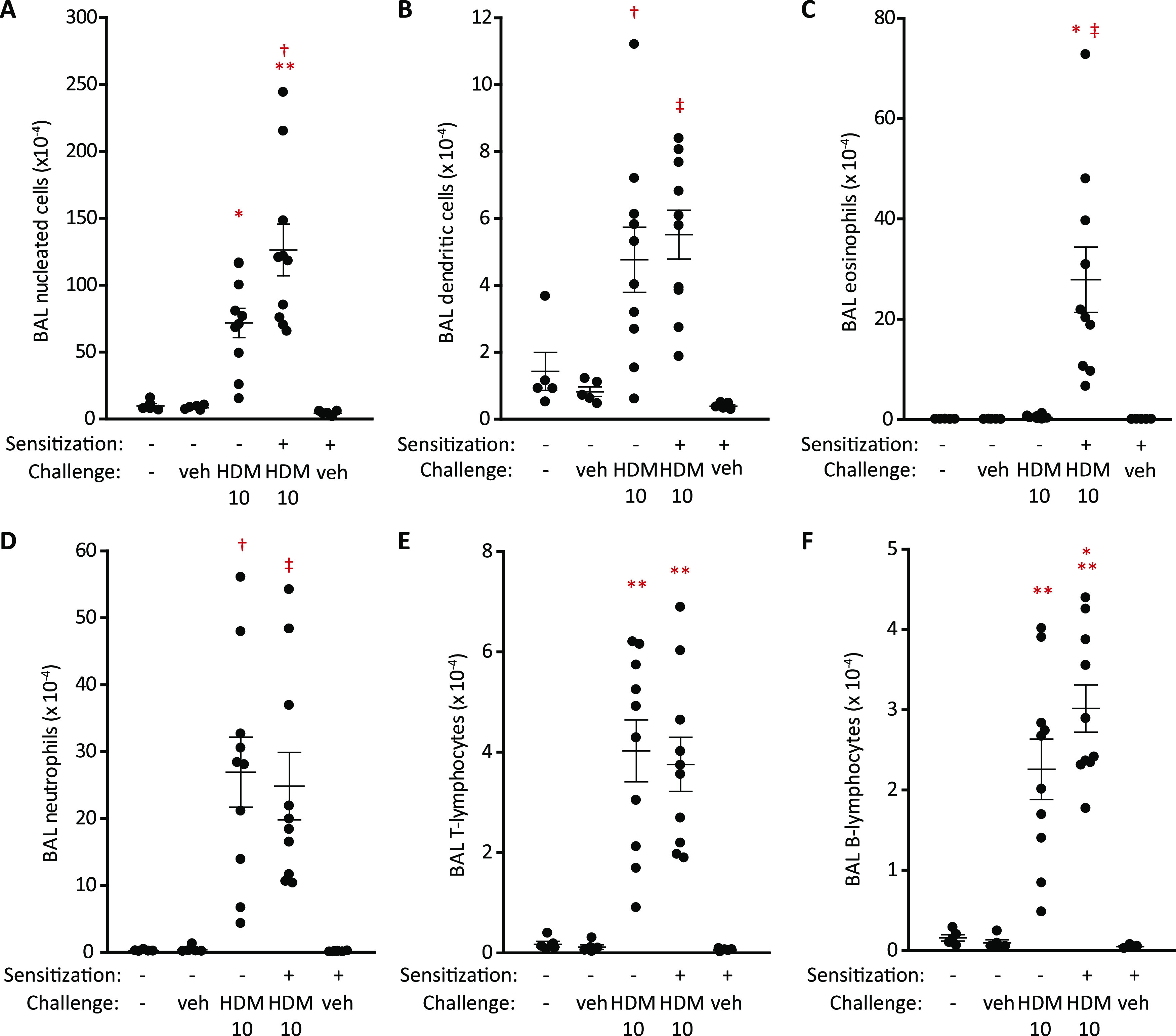
Comparison of IgE-independent and IgE-dependent responses in unsensitized and HDM-sensitized Balb/c mice. (A–F) Flow cytometric analysis of cell counts 48 h following i.t. aerosol allergen challenge (HDM 10, equivalent to 10 μg Der p 1; i.t. aerosol). Data are individual responses with mean ± S.E. depicted by whiskers. In A, *P < 0.05, ^**^P < 0.001 *vs* vehicle (veh) challenge in nonsensitized and sensitized animals; ^†^*P* < 0.01 *vs* HDM challenge in nonsensitized animals. In B, ^†^*P* < 0.01–0.05, ^‡^*P* < 0.001–0.01 *vs* vehicle challenge in nonsensitized and sensitized animals. In C, *P < 0.001 *vs* vehicle challenge in nonsensitized and sensitized animals. ^‡^*P* < 0.01 *vs* HDM challenge in nonsensitized animals. In D, ^†^*P* < 0.01–0.05, ^‡^*P* < 0.001–0.01 *vs* vehicle challenge in nonsensitized and sensitized animals. In E, ^**^P < 0.001 *vs* vehicle challenge in nonsensitized and sensitized animals. In F, ^**^P < 0.001 *vs* vehicle challenge in nonsensitized and sensitized animals. *P < 0.05 *vs* HDM challenge in nonsensitized animals.

To further understand the role of Der p 1 proteolytic activity in driving innate and acquired responses, we next conducted studies in mice. Mice developed sensitization to the HDM extract (elevated total IgE, HDM-specific IgE, and HDM-specific IgG_1_) and allergic responsiveness (Supporting Information Figures S4 and S5). The aerosol challenge evoked a time-dependent increase in BAL fluid cellularity that, like rats, was characterized by a rapid increase in neutrophils (Figure 6). In contrast, elevations in other cells (MHC II^+^, CD11c^+^ DCs; SSC^high^, CCR^3+^, moderate CD11c^+^ eosinophils; macrophages; T- and B-lymphocytes) were slower in onset and sustained (Figure 6). BAL sampling 48 h after the HDM extract challenge was chosen for pharmacological studies, although like rats, this time was suboptimal for neutrophils. Except for eosinophils, the relationship between HDM challenge and cell recruitment had a low dynamic range (Figure 6H–M). Comparison of responses to HDM challenge in sensitized and naive mice (Figure 7) showed that whereas eosinophil recruitment was significantly greater after sensitization (Figure 7C), the accumulation of other cells was like the response of HDM-naive mice (Figure 7B,D–F). This suggests that inhibitory effects on key innate effectors are the predominant mechanism of ADIs, and this could be significant in how the specific inhibition of a single initiator allergen target can affect responses to the HDM allergome generally.

### ADIs Inhibit the Accumulation of DCs, B-Lymphocytes, and Eosinophils

**Figure 8:**
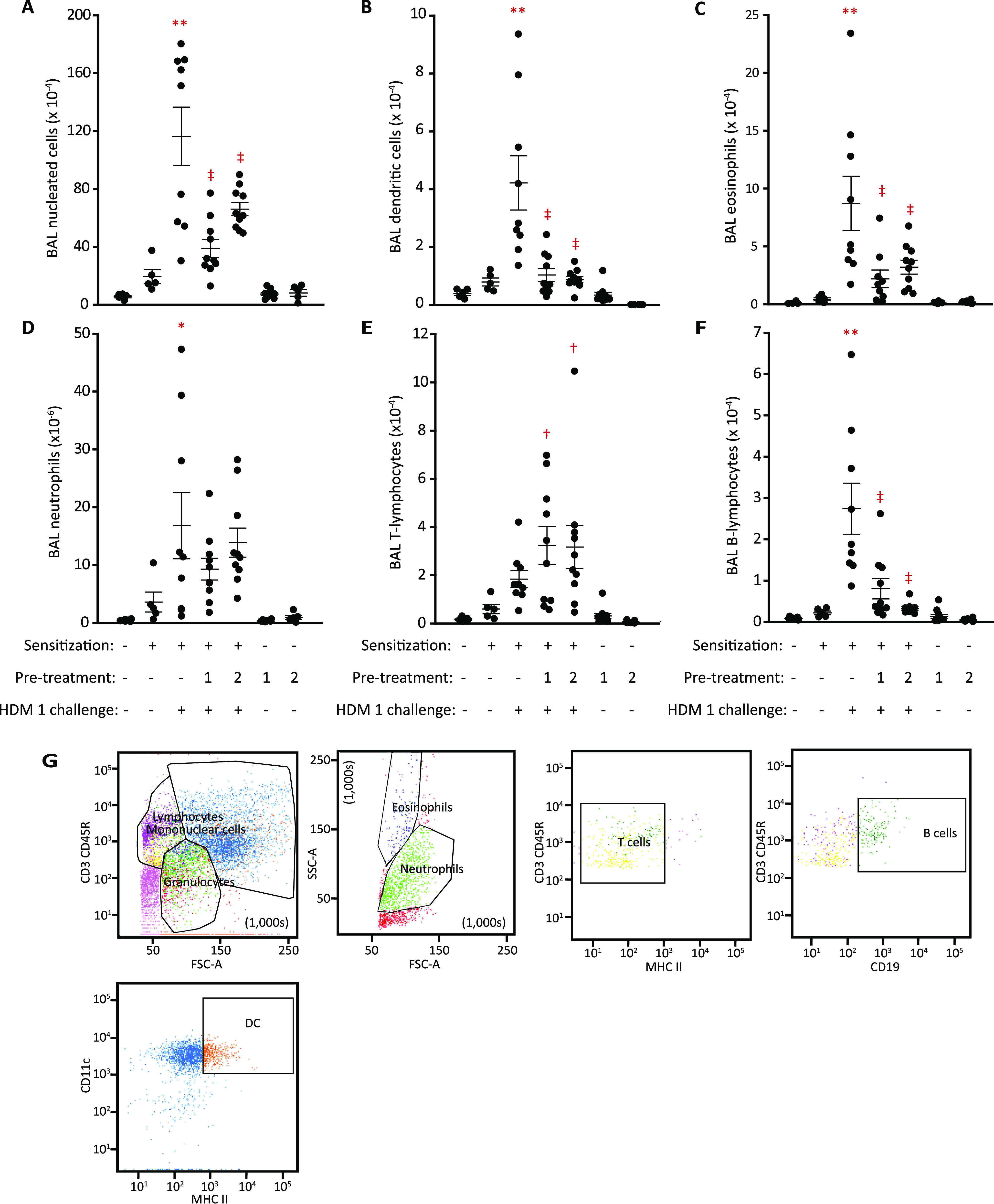
Effects of compounds **1** or **2** following challenge of Balb/c mice with the HDM allergen extract. (A–G) Flow cytometric analysis of cell counts 48 h following i.t. aerosol allergen challenge (HDM 1, equivalent to 1 μg Der p 1; i.t. aerosol). Animals were pretreated with either compound **1** (45 μg/kg) or compound **2** (130 μg/kg) 1 h prior to the HDM challenge. Data are individual responses with mean ± S.E. depicted by whiskers. (G) Flow cytometry profiles in a mouse challenged with the HDM allergen extract (HDM 1). In A–C and F, ^**^P < 0.001 *vs* unchallenged nonsensitized and sensitized mice; ^‡^*P* < 0.001 *vs* control HDM challenge. In D, *P < 0.05 *vs* unchallenged nonsensitized and sensitized mice. In E, ^†^*P* < 0.01 *vs* control HDM challenge.

**Figure 9:**
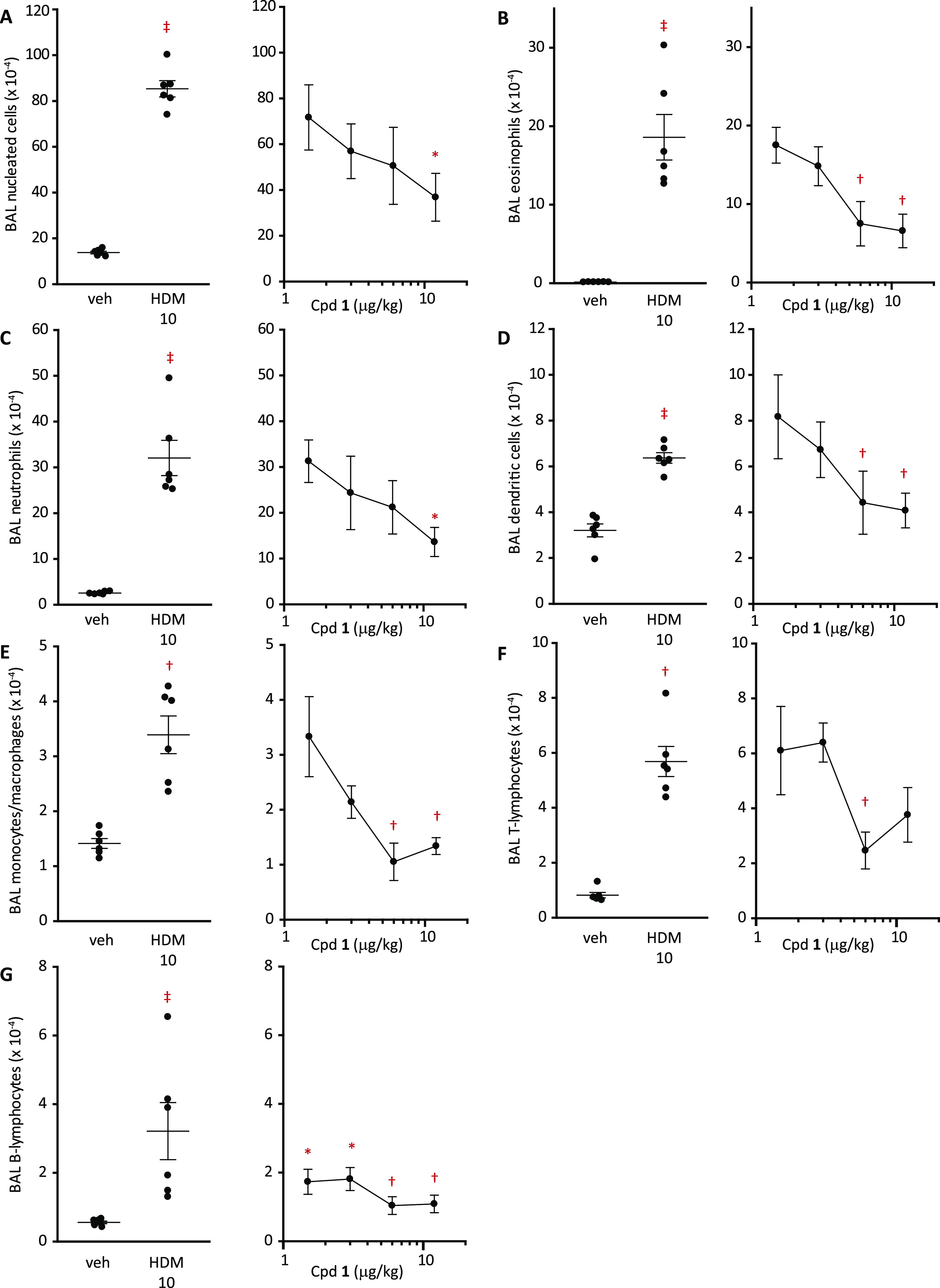
Concentration-dependent inhibition of HDM extract responses in sensitized Balb/c mice by ADI compound **1**. (A–G) Flow cytometric analysis of BAL cells 48 h after challenge with the HDM extract (HDM 10, equivalent to 10 μg Der p 1; i.t. aerosol). Animals were treated i.t. with compound **1** 2 h prior to the HDM challenge. Data are mean ± S.E. from six animals. The effects of the HDM challenge were significant compared to vehicle (veh)-challenged animals. ^‡^*P* < 0.001 in A–D and G; ^†^*P* < 0.01 in E and F. In the dose–response curves, significant effects are denoted as *P < 0.05 and ^†^*P* < 0.01.

In sensitized mice, both compounds strongly suppressed the recruitment of MHC II^+^, CD11c^+^ DCs, and B-lymphocytes and SSC^high^, CCR3^+^, and CD11c^+^ eosinophils by HDM extracts (Figure 8). In contrast, T cells were significantly elevated compared with the control allergen challenge (which itself was not significantly different to the unchallenged control and thus consistent with the data in Figure 6 for the HDM 1 challenge dose), but the characteristics of the cells underlying this response have not been investigated. We next examined the dose–response relationship between compound **1** and BAL composition and found that doses >10 μg/kg were required for good activity against the HDM extract concentration used in these experiments (Figure 9). As the stoichiometry of drug and target will be an important factor in inhibition and given that natural exposures to HDM allergens will be at lower levels than used in our preclinical models, these data provide encouragement that effective and long-lasting inhibition could be achievable at doses compatible with delivery devices in common clinical usage.

Taken together, these preclinical studies demonstrate the feasibility of a small-molecule approach to allergy where the therapeutic target is an apex trigger of the disease. We sought to examine whether it was possible to design pharmaceutically developable ADI NMEs that could provide durable protection against an allergen extract representing the HDM allergome. We evaluated these ADIs in an acute setting with this challenge because it provides a demanding test of the at-source, apex intervention principle. Strikingly, ADI NMEs selective for group 1 allergens were found to inhibit both innate (IgE-independent) and acquired (IgE-dependent) cell and mediator responses to HDM challenge.

Both ADI NMEs attenuated IgE-dependent and IgE-independent events *in vivo* with a similarity, suggesting that their benefits derive from local effects in the airways rather than systemic actions that would be denied compound **2**. In situations where epithelial permeability may be increased,^28,29^ both compounds might obtain systemic exposure, but inspection of their property profiles suggests that this would be tempered by protein binding; a modest half-life; and, for quaternary amines, an exclusion from cellular access contributing to a low volume of distribution.

ADIs are likely to influence events in a range of cell types activated by the HDM allergome. Inhibition of innate responses in airway epithelial cells has already been described by us,^5,19^ while other effects reported here are consistent with an IgE-independent component to degranulation in mast cells^32^ and the upregulation of inflammatory genes and FcεRI in mast cells by innately derived IL-4 and IL-13.^33^ Notable features of ADIs were reductions in eosinophil, DC, and B-lymphocyte numbers. Because of the strong association between eosinophils and Th2-mediated allergic events in humans (including the development of persistent airflow obstruction)^34^ and the linkage between DCs and eosinophils,^35^ our studies sampled at times suited to the dynamics of these cells, but earlier snapshots revealed some suppression of neutrophil responses too.

While being primarily focused on drug design considerations for proof of principle, our data show a reduction *inter alia* in BAL and serum IL-13, which is compatible with an anti-Th2 mechanism exerted by the apex intervention, together with the inhibition of chemokines that activate DCs and lymphocytes. The inhibition of IL-13 may contribute to the suppression of eosinophils by reducing IL-13R-linked, Janus kinase-dependent chemokine production.^36^ Further contributions to eosinophil suppression may arise because ADIs are known to prevent IL-4-dependent IgE class switching^6^ and because disruption of signaling through IL-4 and IL-13 by antibody blockade is established as being clinically effective in reducing eosinophil recruitment.^33^ CCL2 and CCL20, which recruit DCs, basophils, and Th17 cells,^37,38^ were elevated after HDM challenge and suppressed by ADIs. In mice, the HDM extract increased the numbers of DCs in BAL regardless of the sensitization status, and ADIs inhibited this sentinel event. The mechanism(s) accounting for the effects of ADIs on CCL2 and CCL20 has(have) not been established, but ADAM 10, which is activated by Der p 1 in human airway epithelial cells,^5^ is known to be involved in CCL20 release.^39^ Numbers of B-lymphocytes in BAL were also increased by the HDM challenge, and this was ADI-sensitive. The combined blockade of IL-4 and IL-13 signaling, or antagonism of IL-4 alone, suppresses both circulatory and tissue resident B-cells following HDM exposure,^33^ suggesting linkage of this effect of ADIs to decreased cytokine production.

Group 1 HDM allergens trigger the canonical activation of PAR-1 and PAR-4 by thrombin,^19^ leading to EGFR-dependent ATP release, TLR4 ligation, and the generation of ROS.^1,5,9^ This sequence is preventable by ADIs.^5^ As ATP and ROS regulate cytokine gene expression and IL-33 release,^40−42^ this appears to be a crucial axis in the disease because IL-33 exerts IL-13-dependent control over the interactions of epithelial cells with ILC2 cells, innately responsive Th2 cells, and activated DCs.^43^ Notably, post-HDM challenge IL-33 levels in BAL were reduced by ADIs, as were levels of TSLP. This inhibition is interesting considering the reciprocity between their release from the lung and receptor expression in ILC2 cells, their activation of IL-13 release from ILC2 cells, and the ability of both cytokines to directly activate mast cells.^38,44,45^ Collectively, the indications from these studies are that an advantage of ADIs could be the circumvention of mediator redundancy that has been problematic in the development of monoclonal antibody therapies targeting specific cytokines in allergic disease.

Whereas these investigations have focused on using single doses of ADI compounds to demonstrate the intervention principle, we envisage that, in clinical practice, they would be administered chronically where additional benefits might emerge. Preclinically, chronic models have limitations and poorly reflect important disease features relevant to patients (*viz.,* spontaneous airflow limitation and exacerbations) and are thus unreliable predictors of such efficacy gains. Aside from structural differences of the mouse lung, while murine eosinophils exhibit allergen-dependent recruitment to the airways reminiscent of human asthma, they differ in their propensity to degranulate, with potential implications for understanding how ADIs might modify chronic diseases in humans.^46^ Furthermore, the tempo of real-life exposure to HDM allergens is different: smaller amounts over longer periods than typically modeled under laboratory conditions. This difference is helpful from a drug dosing perspective and encourages an exploration of the true promise of this new approach in a clinical setting.

## Experimental Section

### Materials

Media for tissue culture and general laboratory reagents were obtained from ThermoFisher (Paisely, Renfrewshire, UK), Sigma-Aldrich (Poole, Dorset, UK), LGC (Teddington, Middlesex, UK), and GE Healthcare (Little Chalfont, Bucks., UK). Other materials were sourced as indicated.

The Der p 1 assay substrate ((3*S*,6*S*,9*S*,12*S*,15*S*,18*S*)-1-(2-aminophenyl)-9-butyl-18-carbamoyl-15-(4-hydroxy-3-nitrobenzyl)-12-(hydroxymethyl)-3-isopropyl-6-methyl-1,4,7,10,13,16-hexaoxo-2,5,8,11,14,17-hexaazaicosan-20-oic acid (ADZ 50,059)) was synthesized as described previously.^7^ Compound **1** is *N*-{(*S*)-1-[(*S*)-1-((*S*)-1-benzylaminooxalyl-2-methyl-propylcarbamoyl)-ethylcarbamoyl]-2,2-dimethyl-propyl}-benzamide. Compound **2** is 4-{(*S*)-1-[(*S*)-1-((*S*)-1-benzylaminooxalyl-2-methyl-propylcarbamoyl)-ethylcarbamoyl]-2,2-dimethyl-propylcarbamoyl}-1,1-dimethyl-piperidinium formate.

Comparator compounds were as follows: **3** (*N*-[(*S*)-1-((*S*)-1-{(*S*)-1-[2-(3,4-dihydro-1*H*-isoquinolin-2-yl)-2-oxo-ethylaminooxalyl]-2-methyl-propylcarbamoyl}-ethylcarbamoyl)-2,2-dimethyl-propyl]-benzamide); **4** (4-(2-{(*S*)-3-[(*S*)-2-((*S*)-2-benzoylamino-3-phenyl-propionylamino)-propionylamino]-4-methyl-2-oxo-pentanoylamino}-acetyl)-1,1-dimethyl-piperazin-1-ium formate); **5** (quinoline-4-carboxylic acid {(*S*)-1-[(*S*)-1-((*S*)-1-benzylaminooxalyl-2-methyl-propylcarbamoyl)-ethylcarbamoyl]-2,2-dimethyl-propyl}-amide); **6** (N-((*S*)-1-(((*S*)-1-(((*S*)-1-(cyclohexylamino)-4-methyl-1,2-dioxopentan-3-yl)amino)-1-oxopropan-2-yl)amino)-3,3-dimethyl-1-oxobutan-2-yl)isoquinoline-4-carboxamide); **7** (3-((3*S*,6*S*,9*S*)-3-benzyl-9-isopropyl-6-methyl-1,4,7,10,11-pentaoxo-1-phenyl-2,5,8,12-tetraazatridecan-13-yl)benzoic acid); **8** (*N*-((*S*)-1-(((*S*)-1-(((*S*)-4-methyl-1-((2-(4-methylpiperazin-1-yl)-2-oxoethyl)amino)-1,2-dioxopentan-3-yl)amino)-1-oxopropan-2-yl)amino)-1-oxo-3-phenylpropan-2-yl)-1-naphthamide); **9** (4-[((*S*)-3-{(*S*)-2-[(*S*)-3,3-dimethyl-2-(1-oxo-1,3-dihydro-isoindol-2-yl)-butyrylamino]-propionylamino}-4-methyl-2-oxo-pentanoylamino)-methyl]-*N*-methyl-benzamide); **10** (quinoline-4-carboxylic acid [(*S*)-1-((*S*)-1-{(*S*)-1-[2-(4-isopropyl-piperazin-1-yl)-2-oxo-ethylaminooxalyl]-2-methyl-propylcarbamoyl}-ethylcarbamoyl)-2,2-dimethyl-propyl]-amide); **11** (*N*-((*S*)-1-(((*S*)-1-(((*S*)-1-(benzylamino)-4-methyl-1,2-dioxopentan-3-yl)amino)-1-oxopropan-2-yl)amino)-1-oxo-3-phenylpropan-2-yl)isonicotinamide); **12** (*N*-{(*S*)-1-[(*S*)-1-((*S*)-1-cyclohexylaminooxalyl-2-methyl-propylcarbamoyl)-ethylcarbamoyl]-2,2-dimethyl-propyl}-isonicotinamide); **13** (*N*-((*S*)-1-{(*S*)-1-[(*S*)-2-methyl-1-(2-morpholin-4-yl-2-oxo-ethylaminooxalyl)-propylcarbamoyl]-ethylcarbamoyl}-2-phenyl-ethyl)-benzamide); **14** (2-(((*S*)-3,3-dimethyl-1-(((*S*)-1-(((*S*)-4-methyl-1-((2-(4-methylpiperazin-1-yl)-2-oxoethyl)amino)-1,2-dioxopentan-3-yl)amino)-1-oxopropan-2-yl)amino)-1-oxobutan-2-yl)carbamoyl)benzoic acid); **15** (*N*-[(*S*)-adamantan-1-yl-((*S*)-1-{(*S*)-2-methyl-1-[2-(4-methyl-piperazin-1-yl)-2-oxo-ethylaminooxalyl]-propylcarbamoyl}-ethylcarbamoyl)-methyl]-benzamide).

Synthetic routes for compounds **6**–**8**, **11**, and **14** are provided in the Supporting Information. Routes for **1**–**5**, **9**, **10**, **12**, **13**, and **15** have been described elsewhere.^3,47^

### Methods

#### Preparation of HDM Allergen and Purification of Der p 1

*Dermatophagoides pteronyssinus* derived from a wild-caught starter population were grown in a continuous solid-phase culture at 25 °C and 75% relative humidity under barrier conditions. The spent culture medium was harvested, and native HDM allergen extracts were prepared using methods known to preserve labile bioactivity. The spent culture medium harvested in this way has been used as feedstock for the purification of a range of HDM allergens and is, to the best of our practical understanding, representative of the allergenic spectrum of HDM with the probable exception of group 13 allergens that are not exported from cells.^8^ The HDM extract was used for the sensitization and challenge in most studies because it is more representative of the material to which the airways are exposed in life than purified allergens. HDM extracts were normalized to the Der p 1 content expressed as μg/mL. The Der p 1 content of extracts was assayed by ELISA (Indoor Biotechnologies, UK). In experiments using HDM extracts containing 1 μg/mL or 10 μg/mL Der p 1, the total protein delivery was 4 and 40 μg/mL, respectively. The Der p 2 content (ELISA) of the HDM extracts was similar to that of Der p 1. The proteolytic activity of Der p 1 was determined using ADZ 50,059 as the substrate.^7^ Batchwise consistency in the activity of Der p 1 delivered to the lungs was ensured by the inclusion of cysteine or dithiothreitol in vehicles used for the administration of HDM extract aerosols. These were also present in control solutions. The endotoxin content of HDM extracts used in these studies was 2.2 ± 0.4 endotoxin units/μg Der p 1 (*n* = 16).

Purified Der p 1 was required for *in vitro* screening work and used also in some *in vivo* studies. To obtain purified Der p 1, Dulbecco’s PBS (2–3 vol) was added to the HDM extract and stirred overnight. Particulate matter was removed by centrifugation (30 min, 24,000*g*, 4 °C), and solid ammonium sulfate was added to the supernatant to achieve 50% saturation in the presence of 1 mM EDTA. Precipitates formed over >2 h, after which the pellets were collected and reconstituted and insoluble matter was removed for chromatography (Äkta Purifier, GE Healthcare, UK). Recursive size exclusion chromatography (HiPrep 16/60 Sephacryl S-200 HR, GE Healthcare, UK, using 0.2 M sodium phosphate containing 0.5 M sodium chloride and 1 mM EDTA, pH 7.4) and polishing using a soybean trypsin inhibitor (SBTI) column were performed, and the final eluate was desalted by Amicon ultrafiltration through a 10 kDa cutoff membrane (Millipore, Bedford MA, USA). The sample was then chromatographed in 20 mM Tris–HCl buffer, pH 8.0, on Resource Q (GE Healthcare) with Der p 1 being eluted by 0–0.5 M NaCl. Peaks containing Der p 1 were analyzed by SDS-PAGE and MALDI-TOF mass spectrometry (Kratos Axima, Kratos Analytical, UK, or Bruker Flex, Bruker, UK) and combined. Der p 1 was quantified by ultraviolet absorbance in a quartz cuvette at 280 nm (ε = 47,705 M^–1^ cm^–1^). Enzymatic activity was quantified as described below. Purified Der p 1, prepared without specific steps to reduce endotoxin content, contained 0.5–0.7 endotoxin units/μg.

#### Der p 1 Enzyme Activity Assays

Assays were assembled in 96-well plate format using a PerkinElmer Multiprobe II Plus HTS EX robot (PerkinElmer, UK). Reaction mixtures comprised a reaction buffer (70 μL potassium phosphate buffer, pH 8.25, containing 1 mM EDTA), substrate (10 μL at 12.5 μM final concentration), and dithiothreitol (DTT, 10 μL with a final concentration of 1 mM). Reactions were initiated by the addition of 10 μL Der p 1 dissolved in the reaction buffer at 2.5 μg/mL and followed at 30 °C by measurement of fluorescence (excitation/emission 330/420 nm) using either a Fusion Alpha-FP or Envision plate reader fitted with a temperature-controlled carrier (PerkinElmer, UK).

#### Analysis of Inhibitor Kinetics

Inhibitor kinetics were analyzed from progress curves. For reversible inhibitors, IC_50_ values were calculated conventionally.

#### Studies Performed *In Vivo*

Animal studies had ethical review by the institutional care and use committees at AAALAC-accredited contract research partners (Aptuit, Eurofins Panlabs, and Shanghai Chempartner) and were compliant with the Animals (Scientific Procedures) Act (UK) and ARRIVE guidelines. Acute tolerability tests on compounds prior to study commencement did not reveal any adverse events over a 24 h period following dosing.

#### Allergic Responses in Rats

BN rats (male, 250–350 g, Charles River) were housed under isolator conditions and randomly assigned to treatment groups. Sensitization to HDM allergen extract was performed on days 0, 7, and 14 by intraperitoneal (i.p.) injection (0.5 mL). Control animals received saline vehicle treatment.

In routine studies of allergen-induced leukocyte accumulation, rats were briefly anesthetized (isoflurane in oxygen) on day 21, and the vehicle, HDM allergen extract, or HDM allergen extract with ADI compound was delivered from a Penn-Century IA-1C/FMJ-250 aerosolizer. For the duration of protection studies, the dosing of the vehicle or drug was separated from the allergen challenge by predetermined intervals. Animals were allowed to recover from the anesthetic to enable assessment of cell recruitment to the lungs 48 h after the challenge or according to study design. Animals were euthanized with pentobarbitone (250 mg/kg i.p.), and the lungs were lavaged via a tracheal cannula using 3 × 4 mL aliquots of Hanks’ balanced salt solution (HBSS) containing 10 mM EDTA and 25 mM HEPES. Lavaged cells were pooled, and the volume was adjusted to 12 mL with HBSS. Total cells were counted (ADVIA, Bayer Healthcare, Diagnostic Division, UK), and smears were made by diluting the recovered fluid (to ∼10^6^ cells/mL) and pipetting an aliquot (100 μL) into a cytocentrifuge. Air-dried smears were fixed in methanol for 10 s before staining with buffered eosin (10 s) and methylene blue/Azur (5 s) (Speedy-Diff, ClinTech Ltd., UK) to differentiate eosinophils, neutrophils, macrophages/monocytes, and lymphocytes. An independent observer who was unaware of the treatment codings performed the cell counts by light microscopy at ×1000 magnification using an oil immersion objective. For ELISA assays, BAL fluids were centrifuged (400*g*, 5 min 4 °C), and the cell-free supernatants were desalted using PD-10 columns and then freeze-dried pending analysis as outlined in the Supporting Information.

#### Allergic Sensitization Studies in Mice

Mice (female Balb/c 20 ± 2 g, Charles River) were isolator maintained in ventilated cages (Allentown IVC Racks, 36 Mini Isolator System, USA) that had been prepared for use by prior autoclaving. Environmental controls were 22–24 °C/60–80% relative humidity on a 12 h light/dark cycle. Animals were allowed *ad libitum* access to reverse osmosis-purified water and food (MF-18 laboratory rodent diet). Where prestudy serum samples were mandated, these were taken from the retro-orbital sinus on acclimatization in the isolator facility. Animals were randomly assigned to groups and sensitized to the HDM extract or treated with the vehicle on days 0, 7, and 14. Anesthetized animals were challenged by i.t. aerosol on day 21 using a Penn-Century IA-1C/FMJ-250 aerosolizer (20 μL/mouse). Animals were anesthetized with propofol 48 h later (AstraZeneca, 10 mg/mL, 50 μL/mouse, i.v.), and terminal blood samples were taken from the retro-orbital sinus. BAL (3 × 0.5 mL aliquots of PBS) was performed, and the returns were combined for enumeration.

For all *in vivo* studies, the HDM extract or Der p 1 was treated with cysteine to ensure consistent activation. Physiologically, while ASL contains reducing agents able to achieve this and that are known to be elevated in asthma,^13^ the drug discovery campaign required standardized activity through elective *ex vivo* activation. This procedural step has the further benefit of negating variations in activation caused by the dilution of ASL by the aerosol.

#### Flow Cytometry Analysis of BAL Fluid

Flow cytometry (FACS) of BAL fluid was performed with a BD FACSAria instrument (Becton Dickinson Biosciences, USA) and the FACSDiva software. Analyses were performed by operators who were unaware of the sample identity. Unless indicated, antibodies for flow cytometry were obtained from BD Pharmingen (BD Bioscience, Wokingham, Berks., UK): FcγR blocking agent was antibody 2.4G2, MHC class II-FITC conjugate (antibody 2G9), CD11c-allophycocyanin conjugate (antibody HL-3), CD3-phycoerythrin/Cy5 conjugate (antibody 145-2C11), B220 (CD45R)-phycoerythrin/Cy5 conjugate (antibody RA3-6B2), CCR3-phycoerythrin (antibody 83101, R&D Systems, Abingdon, Oxon., UK), and CD19-V450 (rat anti-mouse CD19). Erythrocytes present in BAL were lysed by ammonium chloride, and the nucleated cells were pipetted into 96-well plates. Antibody mix (40 μL in FACS buffer—PBS with 5% w/v BSA and 0.01% NaN_3_—containing antibodies at 2–10 μg/mL) was then added to each well, and labeling was performed in the dark at 4 °C for 30 min. The cells were washed twice in the FACS buffer and resuspended for analysis. Flow cytometry demarcation was as follows: B-lymphocytes FSC^low^/SSC^low^, CD19^+^, CD45R^+^; T-lymphocytes FSC^low^/SSC^low^, CD3^+^; eosinophils SSC^high^, CCR3^+^, moderate CD11c^+^, low-absent MHC II, CD45R/CD3^–^; DCs non-autofluorescent CD3/CD45R^–^, MHC II^+^, CD11c^+^; neutrophils SSC^high^, CCR3^–^, CD11c^–^. Macrophages were distinguished by autofluorescence and size.

#### Data Presentation and Statistical Analyses

Data are shown as mean values ± S.E. Significance was calculated by one-way analysis of variance (ANOVA) with *post hoc* testing using the Student–Newman–Keuls procedure in SigmaPlot v 12.0. A probability value of *P* < 0.05 was considered statistically significant. Relationships between variables were examined using Pearson’s correlation. Sample sizes for *in vivo* studies were determined pragmatically to balance experimental power with ARRIVE/3Rs requirements.

## Supporting Information Available

Development of immune responses in brown Norway (BN) rats treated with house dust mite (HDM) allergen extract and time-dependent changes in bronchoalveolar (BAL) cells following challenge; relationship between cell numbers and cytokine/chemokine levels in BAL fluid from BN rats; duration of protection by pyruvamide scaffold ADI compounds in naive BN rats exposed to i.t. aerosols of HDM allergen extract; pre- and post-immunization serum immunoglobulin concentrations in Balb/c mice; development of cellular responses to the HDM allergen extract in BAL fluid from Balb/c mice; and additional experimental information and methods (PDF)
