# Supplementary material for: Targeting an Initiator Allergen Provides Durable and Expansive Protection against House Dust Mite Allergy
Source: ACS Pharmacol Transl Sci. 2022 Aug 12;5(9):735–51. doi: 10.1021/acsptsci.2c00022 (PMC9469500; doi:10.1021/acsptsci.2c00022)
Supplement: Supplementary file 1 — pt2c00022_si_001.pdf [file pt2c00022_si_001.pdf]

ACS Pharmacology & Translational Science

Supporting Information

Targeting an Initiator Allergen Provides Durable and Expansive Protection Against House Dust Mite Allergy

Jihui Zhang[1a], Jie Chen[1], Jonathan P Richardson[1b], Nicola-Jane Francis-Newton[1], Pei F Lai[1], Kerry Jenkins[2], Meriel R Major[2], Rebekah E Key[2], Mark E Stewart[2], Stuart Firth-Clark[2], Steven M Lloyd[2], Gary K Newton[2], Trevor R Perrior[2], David R Garrod[3], Clive Robinson[1*]

[1]Institute for Infection & Immunity, St George's, University of London, Cranmer Terrace, London SW17 0RE, United Kingdom. [2]Domainex Ltd, Chesterford Research Park, Little Chesterford, Saffron Walden CB10 1XL, United Kingdom. [3]Faculty of Biology, Medicine and Health, University of Manchester M13 9PL, United Kingdom.

*Correspondence: c.robinson@sgul.ac.uk

[a]Present address: State Key Laboratory of Microbial Resources, Institute of Microbiology, Chinese Academy of Sciences, Beijing, P.R. China.

[b]Present address: Centre for Host-Microbiome Interactions, Faculty of Dentistry, Oral & Craniofacial Sciences, King's College London, UK.

This work was supported by the Wellcome Trust (Seeding Drug Discovery Initiative award 087650, to CR).

Supporting Information Figure S1.

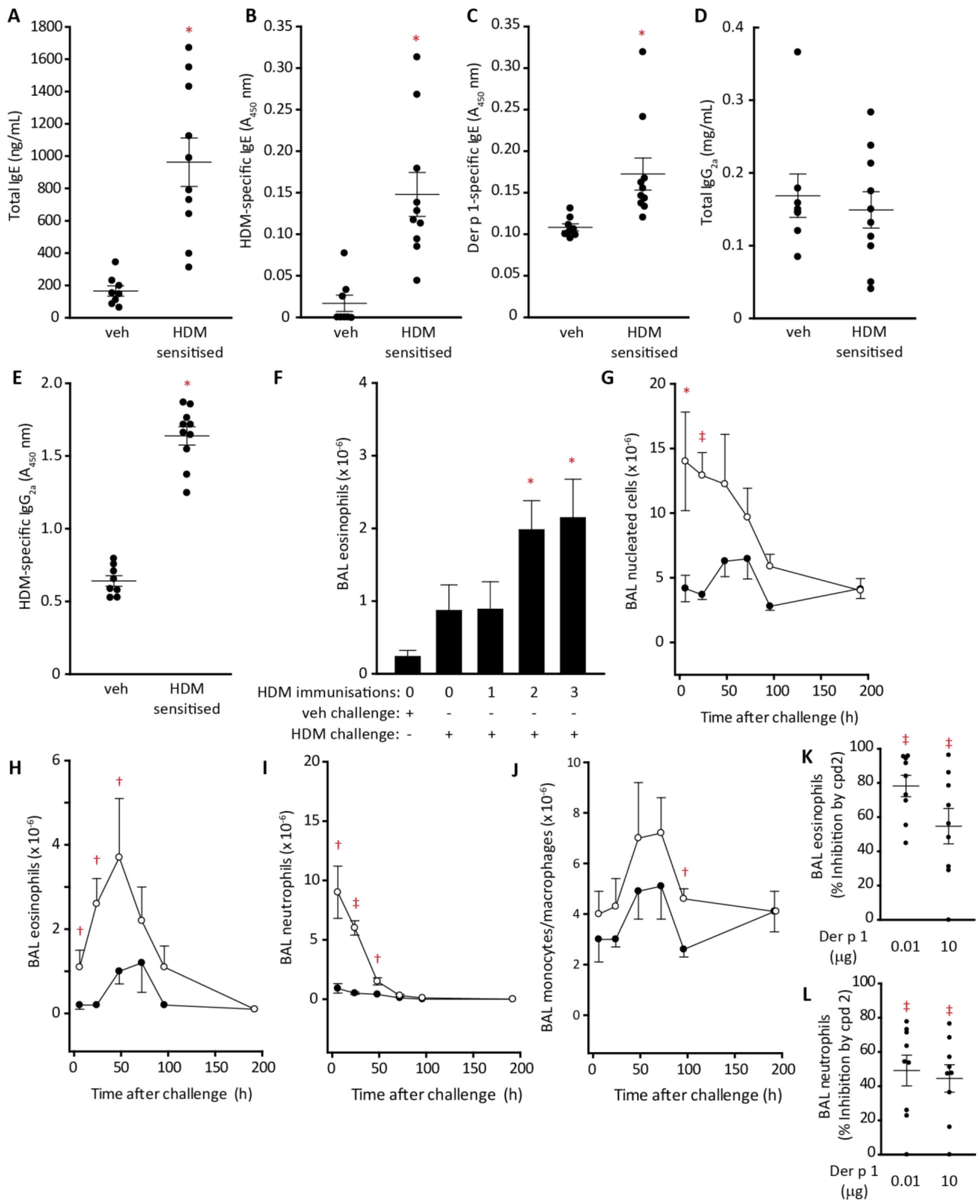

Supporting Information Figure S1. Development of immune responses in brown Norway (BN) rats treated with house dust mite (HDM) allergen extract and time-dependent changes in bronchoalveolar (BAL) cells following challenge.

A Serum IgE concentrations in BN rats sensitised to HDM allergen extract or treated with vehicle (veh); *P<0.001 *vs* veh.

B HDM-specific IgE in serum. *P<0.001 *vs* veh.

C Der p 1-specific IgE. *P<0.01 *vs* veh.

D Total serum IgG_{2a}.

E HDM-specific IgG_{2a}. *P<0.001 *vs* veh.

F Changes in BAL eosinophil numbers evoked by i.t. aerosol challenge with HDM allergen extract (Der p 1 content 10 µg) as a function of the number of HDM exposures used to induce sensitisation. *P<0.05 *vs* unsensitised rats challenged with vehicle.

In panels **A-E** responses are shown for individual animals, in **F** bars denote mean of 10 animals. Whiskers depict s.e. in all cases.

G-J Responses to vehicle challenge in unsensitised animals (filled circles) and HDM extract challenge (HDM 10, Der p 1 content 10 µg) in sensitised animals (open circles). Data are shown as mean ± s.e. from 7 animals per group at each time point. *P<0.05, †P<0.01, ‡P<0.001 compared to corresponding vehicle challenge.

K Inhibition of BAL eosinophil response by compound **2** following i.t. aerosol challenge with 0.01 or 10 µg purified Der p 1.

L Corresponding data for BAL neutrophils. ‡P<0.001 *vs* control treatment. Data are shown for individual animals with mean ± s.e. depicted by whiskers.

Supporting Information Figure S2.

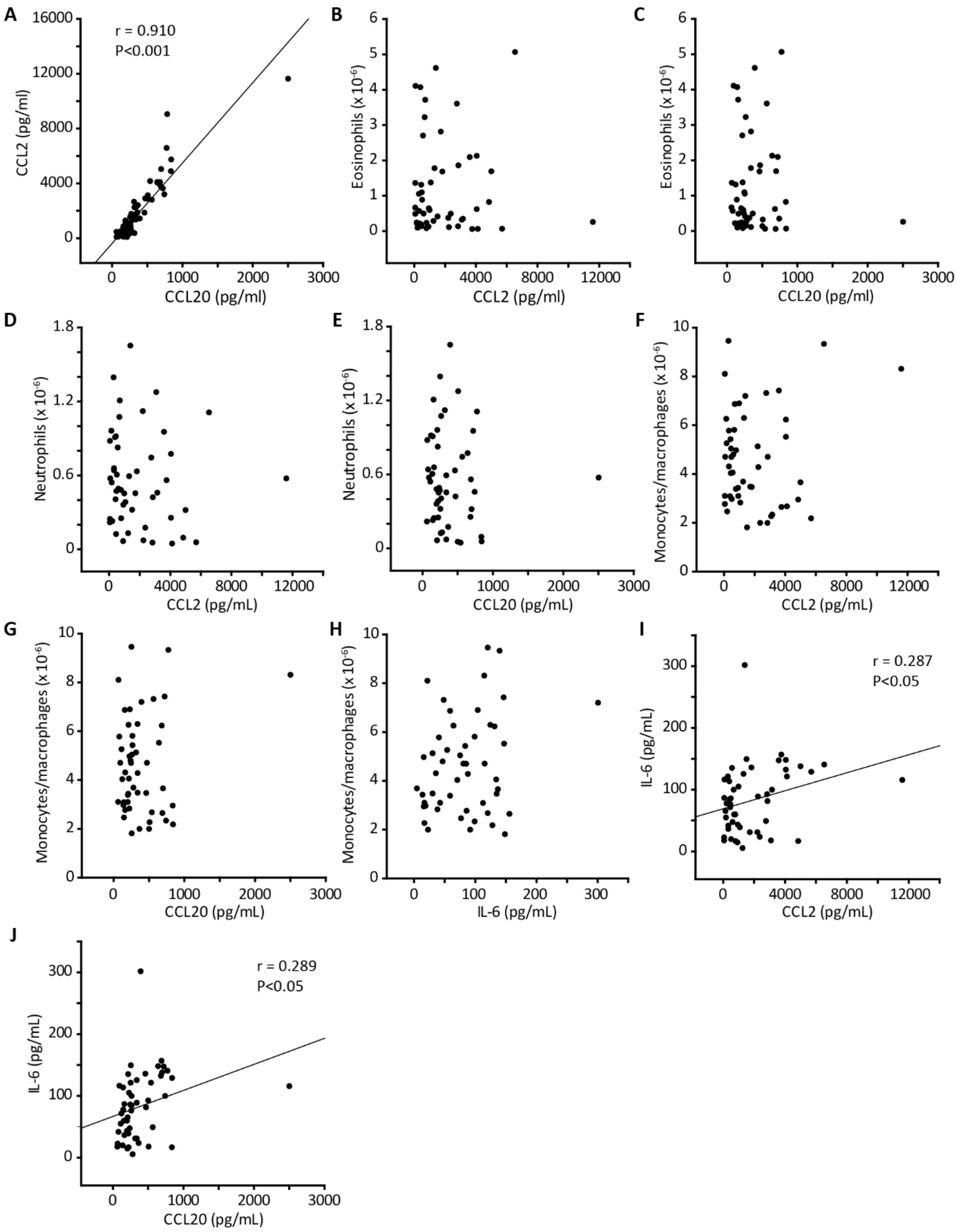

Supporting Information Figure S2. Relationship between cell numbers and cytokine/chemokine levels in BAL fluid from BN rats.

Supporting Information Figure S3.

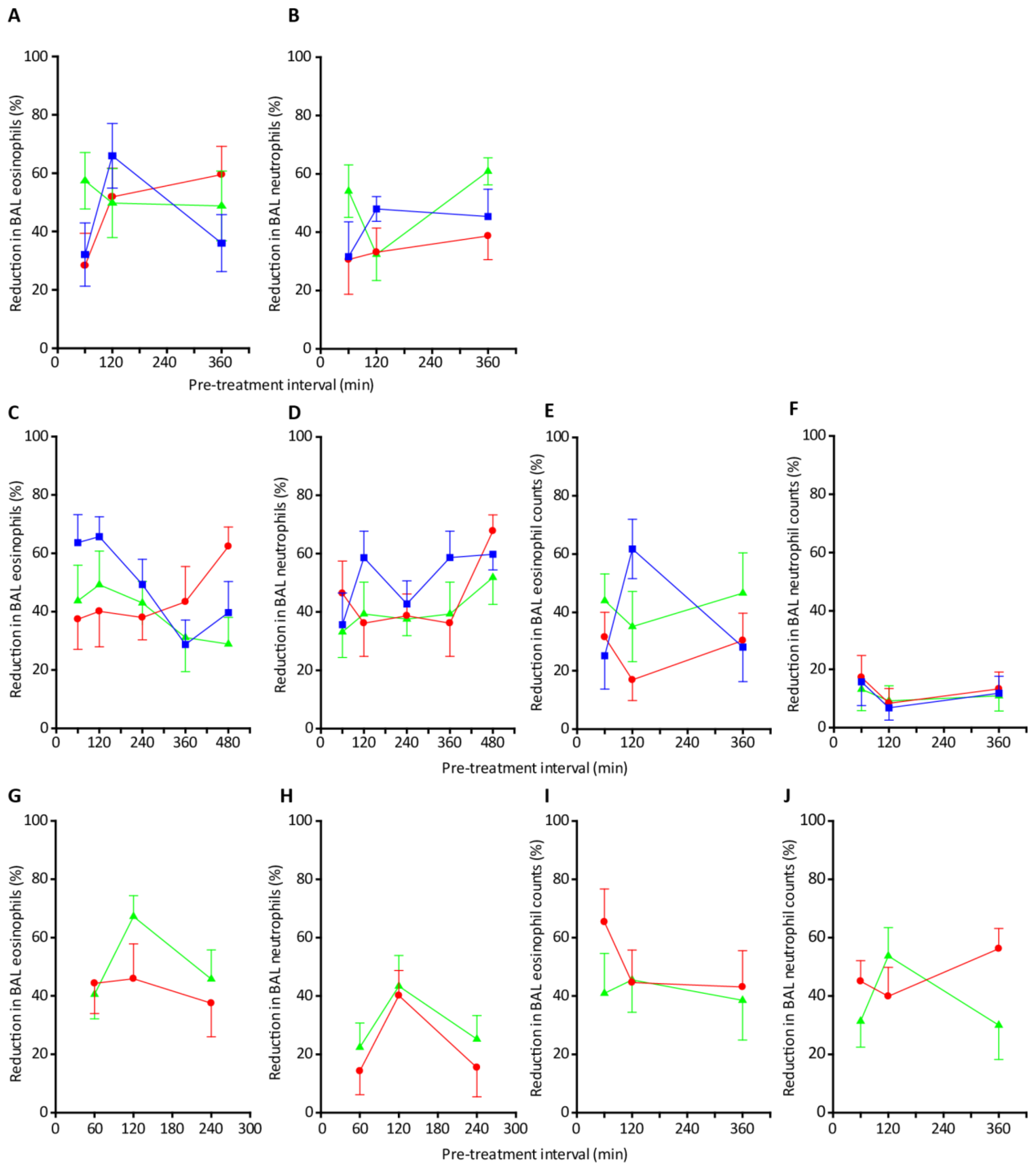

Supporting Information Figure S3. Duration of protection by pyruvamide scaffold ADI compounds in naïve BN rats exposed to i.t. aerosols of HDM allergen extract.

A Reduction in BAL eosinophil response by compounds **3** (red circles), **4** (green triangles) and **5** (blue squares). Inhibition was significant (P<0.05) at all time points for each compound.

B Corresponding data for neutrophils, where all effects were significant (P<0.05).

C Reduction in BAL eosinophil response by compounds **6** (green triangles), **7** (red circles) and **8** (blue squares). Inhibition was significant (P<0.05) at all time points for each compound except compound **6** when pre-treatment was at 360 min or 480 min, and compound **7** at 60 min.

D Corresponding data for neutrophils, where effects were not significant when pre-treatment was at 60 min or 120 min for compound **6**, at 60 min, 120 min or 360 min for compound **7**, and at 60 min for compound **8**, but all other effects were significant (P<0.05).

E Reduction in BAL eosinophil response by compounds **9** (blue squares), **10** (red circles) and **11** (green triangles). Inhibition was significant (P<0.05) at all time points for each compound except compound **9** when pre-treatment was at 60 min or 360 min, and compound **10** at 120 min.

F Corresponding data for neutrophils where the effects were not significant.

G Reduction in BAL eosinophil response by compounds **12** (green triangles) and **13** (red circles). Inhibition was significant (P<0.05) for both compounds at all time points.

H Corresponding data for neutrophils, where the action of compound **13** was not significant when pre-treatment was at 60 min or 240 min, but all other effects were (P<0.05).

I Reduction in BAL eosinophil response by compounds **14** (green triangles) and **15** (red circles). Inhibition was significant (P<0.05) at all time points for both compounds.

J Corresponding data for neutrophils, where inhibition was significant (P<0.05) at all time points for both compounds.

In **A-J**, the delivered Der p 1 content of the HDM extract was 10 µg. In all cases drugs were administered as single doses by i.t. aerosol at the indicated time before HDM extract challenge. Compounds were dosed at a ratio of 50:1 molar equivalents with respect to Der p 1 content of the HDM allergen extract. Data are shown as mean ± s.e. in treatment groups of 10 animals at each time point.

Supporting Information Figure S4.

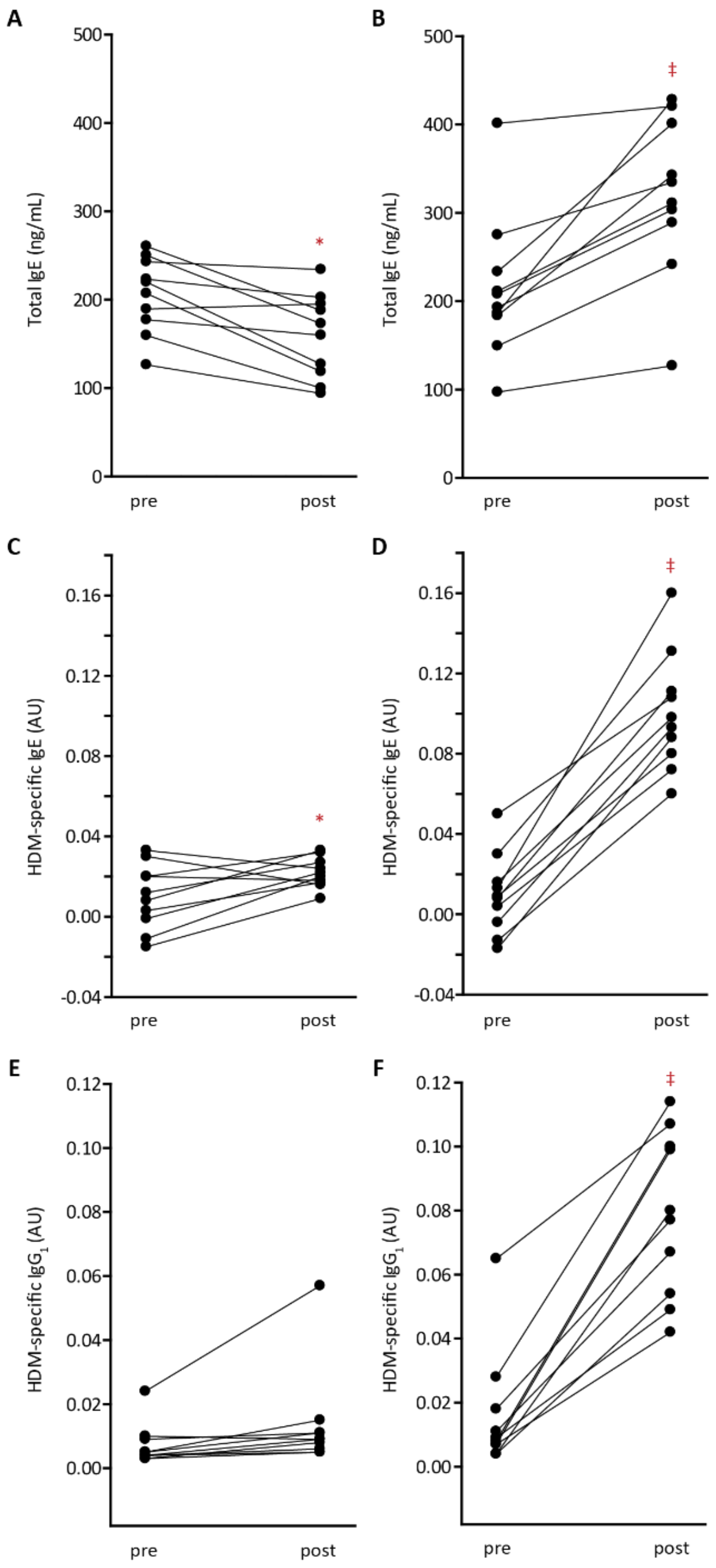

Supporting Information Figure S4. Pre- and post- immunisation serum immunoglobulin concentrations in Balb/c mice.

A Total IgE in mice treated with vehicle.

B Total IgE in mice treated with HDM extract.

C, D Corresponding data for HDM allergen-specific IgE.

E, F Data for HDM allergen-specific IgG_1. Measurements are shown for individual animals before and after immunisation.

$^{*}P<0.05$, $^{‡}P<0.001$ *v* pre-immunisation values.

Supporting Information Figure S5.

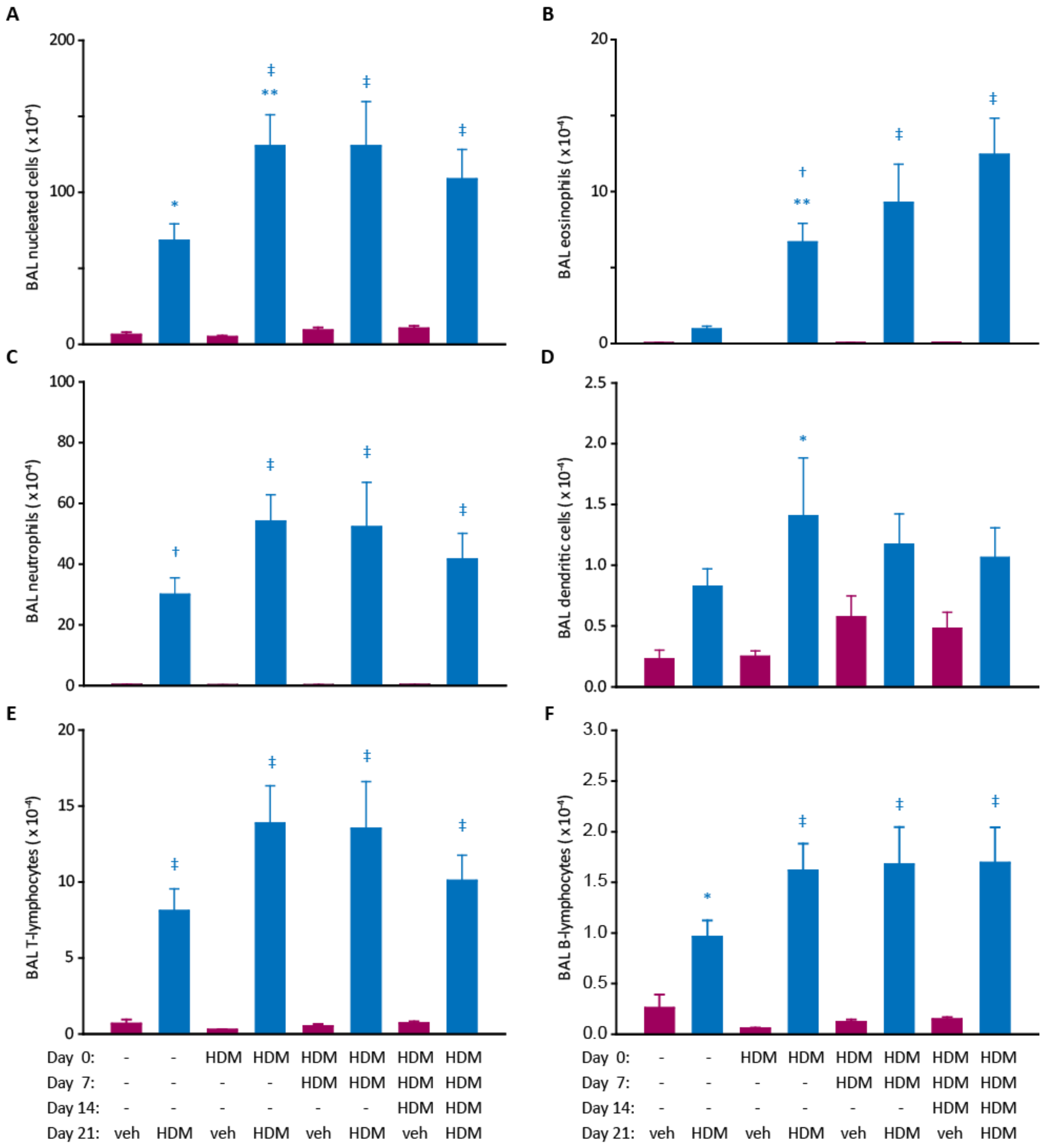

Supporting Information Figure S5. Development of cellular responses to HDM allergen extract in BAL fluid from Balb/c mice.

A-F Results for individual cell types. Blue columns show animals immunised with HDM allergen extract and subsequently challenged with HDM (i.t. aerosol, equivalent to 10 µg Der p 1). Magenta columns depict data for immunised animals challenged with vehicle (veh).

Data are shown as mean ± s.e. in 10 animals per group, $^{*}P<0.05$, $^{\dagger}P<0.01$, $^{\ddagger}P<0.001$ vs corresponding vehicle challenge group. $^{**}P<0.01$-0.05 for mice sensitised on day 0 and challenged with HDM extract on day 21 compared to unsensitised mice challenged with HDM extract on day 21.

EXPERIMENTAL SECTION

Compounds

Compounds **8** and **11** were made by coupling the appropriate β-amino-α-hydroxyamide with the corresponding capped dipeptide using *iso*-butylchloroformate and N-methylmorpholine at low temperature, followed by oxidation of the alcohol with Dess-Martin periodinane to give the desired pyruvamide.

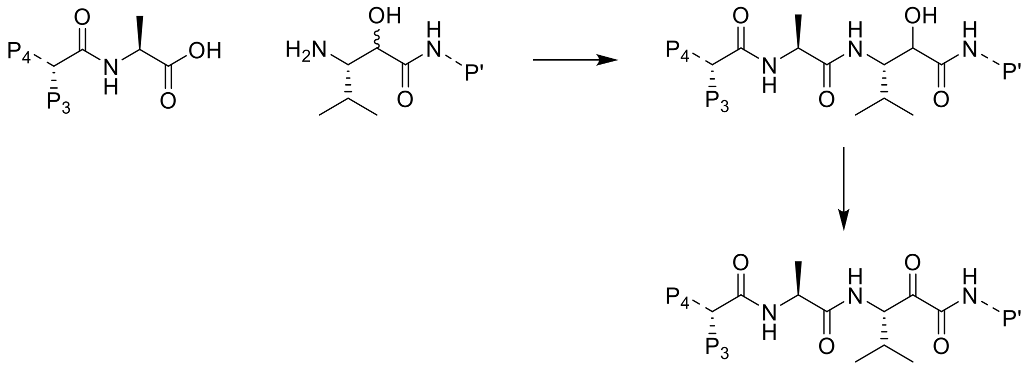

Compounds **6** and **14** were made in a similar manner, however in these instances the carboxylic acid groups were protected as esters which were subsequently hydrolysed in an additional step to yield the target compounds. In the case of compound **6** a methyl ester was used, which was hydrolysed using lithium hydroxide. In the case of compound **14** a *tert*-butyl ester was used which was converted to the carboxylic acid using trifluoroacetic acid in the presence of tri-isopropylsilane.

Compound **7** was made from the corresponding aldehyde using a modified Passerini reaction followed by oxidation of the hydroxyamide using Dess-Martin periodinane.

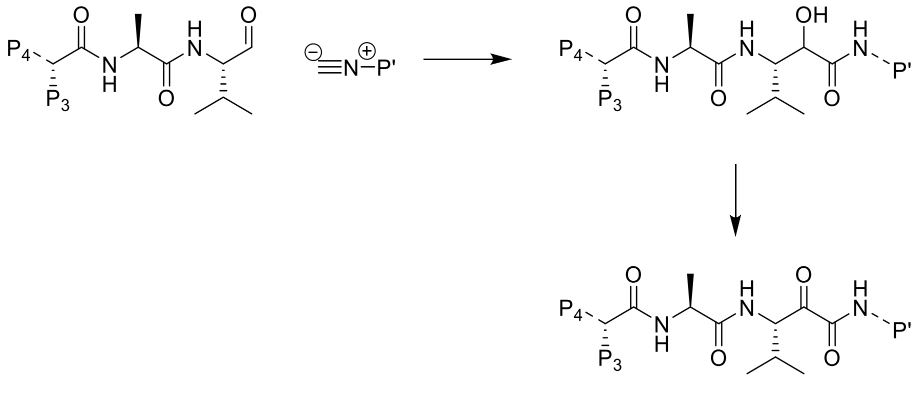

Full details of the above syntheses are described in a previous paper and in patent application WO2011/089396 and its granted forms in multiple territories [1,1].

Plasma Protein Binding Assay

Plasma protein binding (PPB) was determined using Rapid Equilibrium Dialysis chambers with 8 kDa cut-off inserts (Fisher Scientific UK Ltd, Leicestershire, UK). Rat (Sprague Dawley strain) plasma EDTA K_2 (pooled, mixed sex, SeraLab, West Sussex, UK) was centrifuged (13,000 x *g*, 10 min) to remove precipitates before addition of compounds (initial concentration 5 μM). Concentrations were determined by liquid chromatography-tandem mass spectrometry (LC-MS-MS) (AB Sciex 3200 QTrap) after 4 h incubation at 37 °C with binding being calculated from the concentration of compound in each chamber. Assays were performed in replicate and the data expressed as mean ± s.e.

Cellular Stability of Compounds

The cellular stability of compounds *in vitro* was tested using rat airway epithelial cell line L2 and rat alveolar macrophage line NR8383 (ATCC©CRL-2192™). Assays were performed in replicate and the data expressed as mean ± s.e. mean. L2 cells were cultured to confluence in T25 flasks or 24-well plates using Kaighn's modification of Ham's F12 medium (F12K). Cells were prepared for assay by washing 3 times with serum-free F12K. NR8383 Airway macrophages were grown in suspension culture in F12K, collected by centrifugation, washed in serum-free medium and aliquoted into test plates (1.4×10^6 cells/well). Both cell lines were treated with ADI compounds (100 nM) in serum-free F12K in a 5% CO_2 environment at 37 °C. Samples were removed for quantitative analysis by LC-MS-MS using appropriate multiple reaction monitoring transitions for each analyte.

Measurement of Log $D_{7.4}$

n-Octanol (0.7 mL), pre-saturated with 10 mM PBS, pH7.4, was added to test compound (~1 mg) in a 2 mL HPLC vial followed by PBS (0. 7 mL), which had been pre-saturated with n-octanol. The mixture was shaken for 90 min before centrifugation for 10 min at 4,000 rpm. The upper organic layer (0.2 mL) and the aqueous layer (0.5 mL) were removed for analysis by LC-MS. Peak identity was confirmed by mass spectrometry and concentration assessed spectrophotometrically using a diode array detector. The log

D for each compound was calculated by taking the log of its concentration ratio between the octanol and aqueous layers.

Predicted Log P

Log P values were predicted computationally within ChemBioOffice using fragment-based algorithms developed by the Medicinal Chemistry Project and BioByte.

Endotoxin

Endotoxin content in HDM extract or purified Der p 1 was determined by kinetic chromogenic assays using Endochrome-K™ (Charles River) according to procedures provided by the manufacturer. Endotoxin content of HDM extracts used in these studies was 2.2 ± 0.4 endotoxin units/µg Der p 1 (n=16). For reference, analysis of a commercial HDM extract yielded estimates of 116-141 endotoxin units/µg Der p 1. Purified Der p 1, prepared without specific steps to reduce endotoxin content, contained 0.5-0.7 endotoxin units/µg.

ELISA Measurements

Cytokines and chemokines were quantified using kits and according to manufacturer protocols (Biosource Europe, Belgium). Der p 1 was quantified by ELISA (Indoor Biotechnologies, UK). Total serum IgE was measured in Corning® Costar® high binding 96-well microplates using a sandwich ELISA. For rat serum, plates were coated overnight with mouse anti-rat IgE (clone B41-1, BD Biosciences Europe) in carbonate-bicarbonate buffer and blocked using DPBS with 0.05 % v/v Tween® 20 (DPBST) containing 10 % v/v foetal bovine serum. Rat serum samples or IgE standards (BD Biosciences) were prepared in Can Get Signal® Solution 1 (2B Scientific, UK) and incubated on the plate for 15 h at 4 °C. After washing, the secondary antibody (biotin-conjugated mouse anti-rat, clone B41-3, BD Biosciences) and streptavidin-horseradish peroxidase were added in Can Get Signal® Solution 2 and the plate incubated at room temperature for 3.5 h. After washing, substrate solution (3,3’,5,5’-tetramethylbenzidine dihydrochloride in phosphate-citrate perborate buffer) was added to each well followed by 2 M sulphuric acid after incubation in the dark for 5-15 min. Plates were read at 450 nm and unknowns quantified from standard curves. A similar procedure was used for the measurement of mouse IgE using an OptEIA™ set (BD Biosciences).

Allergen-specific immunoglobulins were measured by indirect ELISA. After coating with HDM extract overnight at 4 °C in carbonate-bicarbonate buffer and blocking, serum samples were added and incubated before addition of the appropriate rat (RG7/1.30 or B41-3, BD Biosciences) or mouse (BD OptEIA or MCA336P, AbD Serotec, UK) secondary antibodies in Can Get Signal® Solution 2. Assays were developed as above, and data reported as absorbance at 450 nm.

Pharmacokinetics

Soluble formulations were developed and verified by high performance liquid chromatography (HPLC) with UV absorbance detection so that compounds **1** and **2** could be administered discretely i.v. (1 mg/kg, 1 mL/kg) and p.o. (5 mg/kg, 5 mL/kg) in non-fasted Sprague-Dawley rats (male, 200-300 g, 10-16 weeks of age, groups of three animals per route per compound). Timed blood samples were taken after dosing and plasma prepared for LC-MS-MS quantification of the test substance after inclusion of an internal standard. A non-compartmental model in WinNonlin software was used to calculate pharmacokinetic parameters.

References

[1] Robinson, C., Zhang, J., Garrod, D. R., Perrior, T. R., Newton, G. K., Jenkins, K., Beevers, R. E., Kimberley, M. R., and Stewart, M. R. (2011) Pyruvamide compounds as inhibitors of dust mite group 1 peptidase allergen and their use.

This research was funded in whole, or in part, by the Wellcome Trust [087650]. For the purpose of open access, the author has applied a CC BY public copyright licence to any Author Accepted Manuscript version arising from this submission.
